# Conversational AI Phone Calls to Support Patients With Atrial Fibrillation: Randomized Controlled Trial

**DOI:** 10.2196/64326

**Published:** 2025-08-19

**Authors:** Ritu Trivedi, Liliana Laranjo, Simone Marschner, Aravinda Thiagalingam, Stuart Thomas, Saurabh Kumar, Tim Shaw, Clara K Chow

**Affiliations:** 1 Westmead Applied Research Centre, Faculty of Medicine and Health, The University of Sydney, Level 5, Block K, Westmead Hospital, Hawkesbury Road, Westmead, 2145, Australia, 61 2 8890 3125; 2 Cardiology Department, Westmead Hospital, Westmead, Australia; 3 Charles Perkins Centre, Faculty of Medicine and Health, The University of Sydney, Camperdown, Australia

**Keywords:** atrial fibrillation, quality of life, natural language processing, self-management, digital health, randomized controlled trial, conversational artificial intelligence, feasibility, phone calls

## Abstract

**Background:**

Patient education and self-management support are critical for atrial fibrillation (AF) management. Conversational artificial intelligence (AI) has the potential to provide interactive and personalized support, but has not been evaluated in patients with AF.

**Objective:**

This study aimed to evaluate the feasibility of a conversational AI intervention to support patients with AF postdischarge.

**Methods:**

This was a single-blinded, 4:1-parallel–randomized controlled trial with process evaluation of feasibility and engagement. The primary outcome was the change in Atrial Fibrillation Effect on Quality-of-Life (AFEQT) questionnaire total score between groups. Patients with AF (18 y and older) were recruited postdischarge from Westmead Hospital cardiology services and randomized to receive either the intervention or usual care. The 6-month intervention consisted of fully automated conversational AI phone calls (with speech recognition and natural language processing) that regularly assessed patient health and symptoms and provided self-management support and education. These phone calls were supplemented with an online survey (sent via text message or email) containing replicated call content when participants could not be reached after 3 call attempts. If participant responses were concerning (eg, poor overall health, low medication confidence, and high symptom burden), they would be followed up with an ad hoc phone call and directed to clinical care if required. A semipersonalized education website was also available as part of the intervention, and participants were encouraged weekly (nudges delivered via text messages or emails) to visit it.

**Results:**

A total of 103 patients (mean age, 63.7 y, SD 11.2 y; n=72, 70% male) were randomized (82 to the intervention); the target sample size was 385. The difference in the AFEQT total score was nonsignificant (adjusted mean difference 2.08, 95% CI −7.79 to 11.96; *P*=.46). An exploratory prepost comparison revealed an improvement in total AFEQT score in the intervention group only (baseline: 69.9, 95% CI 64.4 to 75.5; 6 months: 79.9, 95% CI 74.9 to 84.8; *P*=.01). Participants completed 4 of 7 outreaches on average, and 88.4% (304/344) of completed outreaches were reported as useful.

**Conclusions:**

This proof-of-concept study demonstrates the feasibility of conversational AI in supporting patients with chronic conditions postdischarge. Intervention participants had improvement in their atrial fibrillation quality of life, though the forced shortening of the evaluation was unable to demonstrate a significant difference between groups.

## Introduction

The increase in atrial fibrillation (AF) prevalence is a global public health concern. AF presents the health system with various challenges: its rapidly increasing patient population [1], the multifaceted care required to manage patients and prevent outcomes of stroke and mortality [2], and the significant costs associated with hospitalizations [3]. Guidelines suggest a digitally enabled integrated approach to AF management, involving a multidisciplinary team to provide patient-centered care and support patient self-management (eg, lifestyle behavior change and medication adherence) [2].

Existing trials of digital interventions to support AF self-management have found mixed results, with some evidence of improvements in quality of life (QoL), knowledge, medication adherence, and clinical outcomes (composite outcome comprising stroke or thromboembolism, all-cause death, and rehospitalization) compared to usual care [4-6]. Most digital interventions for patients with AF have primarily been delivered through mobile apps, and many have reported low user engagement [7-9].

Conversational technologies now offer interactive ways of providing education and self-management support to patients [10]. These technologies can simulate human conversations through text or speech in an accessible and personalized manner, with users reporting high satisfaction. Recent voice-based conversational technologies leverage artificial intelligence (AI; including speech recognition and natural language processing) to facilitate more engaging and human-like dialogues [11,12]. There is limited literature available on the efficacy of conversation-AI interventions, and no studies have been conducted in an AF population [11-14].

The aim of this proof-of-concept randomized controlled trial (RCT) was to evaluate an intervention comprising conversational AI automated phone calls, text messages, and emails, and an educational website to better support patients with self-managing their AF [15]. The Coordinating Health Care With Artificial Intelligence–Supported Technology for Patients With Atrial Fibrillation (CHAT-AF) trial assessed the impact of this conversational AI intervention on Atrial Fibrillation Specific Quality of Life (AF-QoL), as well as evaluated its feasibility (engagement and perceived usefulness).

## Methods

### Study Design

CHAT-AF was a single-blinded parallel RCT, with a 4:1 allocation chosen to optimize process evaluation [15]. Trial registration with the Australian and New Zealand Clinical Trials Registry was submitted on November 25, 2020, but COVID-19 delays led to the trial only being registered on February 18, 2021 (85 d post initial submission; ACTRN12621000174886), and at this time, there were 22 participants enrolled.

### Patient Population

Participants were recruited from inpatient and outpatient cardiology services at Westmead Hospital. English-speaking adult patients with documented AF who had a mobile phone were eligible. Pregnant women and those participating in another clinical trial focused on providing AF education were excluded. Original plans to conduct a multicenter trial did not proceed after news of the acquisition of the technology partner by another organization (details below).

### Randomization and Masking

Randomization was 4:1 (intervention: control), stratified by sex, in blocks of 5. The allocation sequence was incorporated into REDCap (Research Electronic Data Capture; Vanderbilt University) [16] with access groups enabled, where data analysts were blinded. Participants, care providers, and research assistants were not blinded. Participants were randomized to receive usual care or the conversational AI intervention.

### Study Procedures

All participants completed study assessments via electronic surveys at baseline (in hospital or sent a link via text messages or email), 3-, and 6-months (via a link sent by text message or email). Baseline demographics and medical history were collected by participant self-report or by electronic medical records review (Multimedia Appendix 1). Questionnaires to assess primary and secondary outcomes were also completed electronically, either in person (baseline—during initial hospital visit) or sent via text message or email (3- and 6-month follow-up).

### Ethical Considerations

The human and research ethics committee at Western Sydney Local Health District (2020/ETH02546) approved this study. Informed consent, either written or over-the-phone consent, was obtained from all study participants. Data were collected and stored on secure servers accessible to approved study personnel only. Minimal participant data were provided to the technology partner via a secure file transfer protocol to enable delivery of the intervention phone calls, text messages, and emails. All data were deidentified for data analysis and publication. No compensation was offered to participants.

### Intervention (AF-Support)

The CHAT-AF intervention design and development have been previously described in detail elsewhere [15]. In summary, it consisted of 7 outreaches (“digital visits”) and a semipersonalized education website, which was available as part of the 6-month digital intervention (Multimedia Appendix 1). The technology in the intervention was provided by HMS (Health Management Systems, Inc).

The outreaches were delivered via fully automated conversational AI phone calls (with speech recognition and natural language processing capabilities) and supplemented with an online survey (a personalized link sent via text message or email) when participants could not be reached after 3 call attempts. Two main components underpinned the conversational AI in the automated calls [17]: (1) a speech recognition engine that recognized voice responses and translated them into text, and (2) natural language processing that identified the semantic and syntactic elements from user utterances, progressing the flow of the call depending on patient answers, in a decision tree format. Given the proprietary nature of the software and the acquisition of the technology partner by another company, we were unable to obtain details about the network architecture, speech recognition, natural language processing capabilities, and other aspects related to the AI models in this intervention. During the phone calls, patients received AF education (eg, lifestyle information on diet and physical activity, importance of general practitioner [GP] follow-up, medication adherence, alcohol intake, blood pressure control, stroke, sleep apnea, and AF procedures) and were required to verbally respond to risk assessment queries (eg, overall health status, GP follow-up, AF symptoms and impact on daily life, medication confidence, and adherence). Certain patient responses to risk assessment questions would trigger alerts leading to an escalation pathway with clinician support, where needed. For example, if patients reported poor overall health status or significant impact of AF symptoms on daily life, these alerts would be actioned within 24 to 48 hours through a phone call by the researcher and additional clinical follow-up if required.

The 7 digital outreaches were also accompanied by weekly text messages or emails (depending on preferences) to a personalized link to an educational website that was tailored based on baseline characteristics (smoking status, alcohol consumption, hypertension diagnosis, and anticoagulant or warfarin prescription). This website contained AF-related information in the form of videos, texts, and images, as well as external links to online resources from reputable sources (eg, Heart Foundation and National Prescribing Service).

### Modified Delivery of Intervention and Premature Study Stopping

At the end of July 2021, we stopped recruitment as the delivery of the intervention was interrupted due to unforeseeable circumstances involving the acquisition of the technology partner by another organization. The trial steering committee made a decision to continue the trial to ensure the full 6-month program was delivered to all enrolled participants, by enabling feasible delivery of intervention content through text messages and emails. The technology partner had notified the research team in advance, allowing for the opportunity to develop an alternative approach and pre-emptively notify participants of the change. Further, comprehensive reports on each participant’s intervention completion status were made available, which allowed for a more seamless transition period. At this point, 82 intervention participants were recruited, and of these, 20 had received all outreaches, with the remaining 62 being at differing stages of the intervention timeline. All participants had received at least 2 of 7 outreaches via the automated calls before premature study completion. The survey (delivered via REDCap) contained replicated content and questions as asked in the phone calls but required participants to click their responses. The hope had been to find another technology partner that could deliver the intervention according to our specifications, but we were unable to do this, and limited by the remaining budget, we stopped the study to report findings. These changes were planned, reviewed, and approved by the trial steering committee. This trial is reported according to the CONSERVE (CONSORT [Consolidated Standards of Reporting Trials] and SPIRIT [Standard Protocol Items: Recommendations for Interventional Trials] Extension for RCTs Revised in Extenuating Circumstances) statement [18].

### Outcomes

The primary outcome was change in AF-QoL assessed as Atrial Fibrillation Effect on Quality-of-Life (AFEQT) [19] total score at 6 months. AFEQT total score (0‐100) is an average of subscales (symptom, daily activity, or treatment), with higher scores indicating better QoL.

Secondary outcomes included AFEQT subscales, AF knowledge [20], patient activation [21], patient assessment of care quality and self-management support [22], self-reported lifestyle behaviors, medication adherence (days of missed doses in prescription medications over the past week), health care service use (GP or cardiologist visits, emergency department presentation or hospitalization, and ablation or cardioversion procedure), and health outcomes (stroke or myocardial infarction). All outcomes were assessed at baseline and 6 months; AF-QoL (AFEQT) was also assessed at 3 months. A detailed list of outcomes, methods, and time of data collection is provided online (Multimedia Appendix 1).

Process evaluation outcomes for the intervention group included: outreach completion and perceived usefulness, and individual engagement (Multimedia Appendix 1). Outreach completion was calculated as the number of outreaches with at least half of the questions answered divided by the number of participants that received the outreach. Outreach perceived usefulness was defined as the number of individuals who answered, “yes” to the question “Did you find the information in this call/survey helpful?” and was divided by the number of participants who attempted the outreach (answered at least 1 question). Individual engagement was calculated as the number of outreaches completed by the participant and categorized, where 4 or more completed outreaches (of 7) were considered as “higher engagement.” Metrics for engagement with the educational website were also explored.

### Statistical Analysis

A sample size of 385 was required to detect a between-group difference of 7 in the total score of the AFEQT questionnaire with 80% power (*α*=.05; SD=19), accounting for a dropout rate of 10% [19,23]. The current study was limited in detecting differences in primary outcome as we were only able to recruit 27% (103 participants) of the intended sample size.

Analyses were prespecified in a statistical analysis plan (Multimedia Appendix 2 [15,19-22,24]) and were conducted according to intention-to-treat principles. Analyses were performed using R statistical software (version 4.1.2; R Project for Statistical Computing). Outcomes were analyzed using either ANCOVA or logistic regression, adjusting for baseline variables. All tests were 2-tailed, a *P* value of <.05 was considered significant, and odds ratios were reported with 95% CIs. Normally distributed continuous variables were expressed as mean and SD. Nonnormally distributed variables were expressed as the median and IQR.

A univariate logistic regression analysis to predict higher engagement was conducted with covariates of age, gender, ethnicity, education, type of AF, time since AF diagnosis, and CHA_2_DS_2_-VASC score (congestive heart failure, hypertension, age 75 y and older [2 points], diabetes, stroke [2 points], vascular disease, aged 65 to 74 years, and sex category [female]; calculated as a sum, where 1-point or 2-points [where indicated] is given when aforementioned characteristics are present) [24].

## Results

### Sample Characteristics

**Figure 1.**
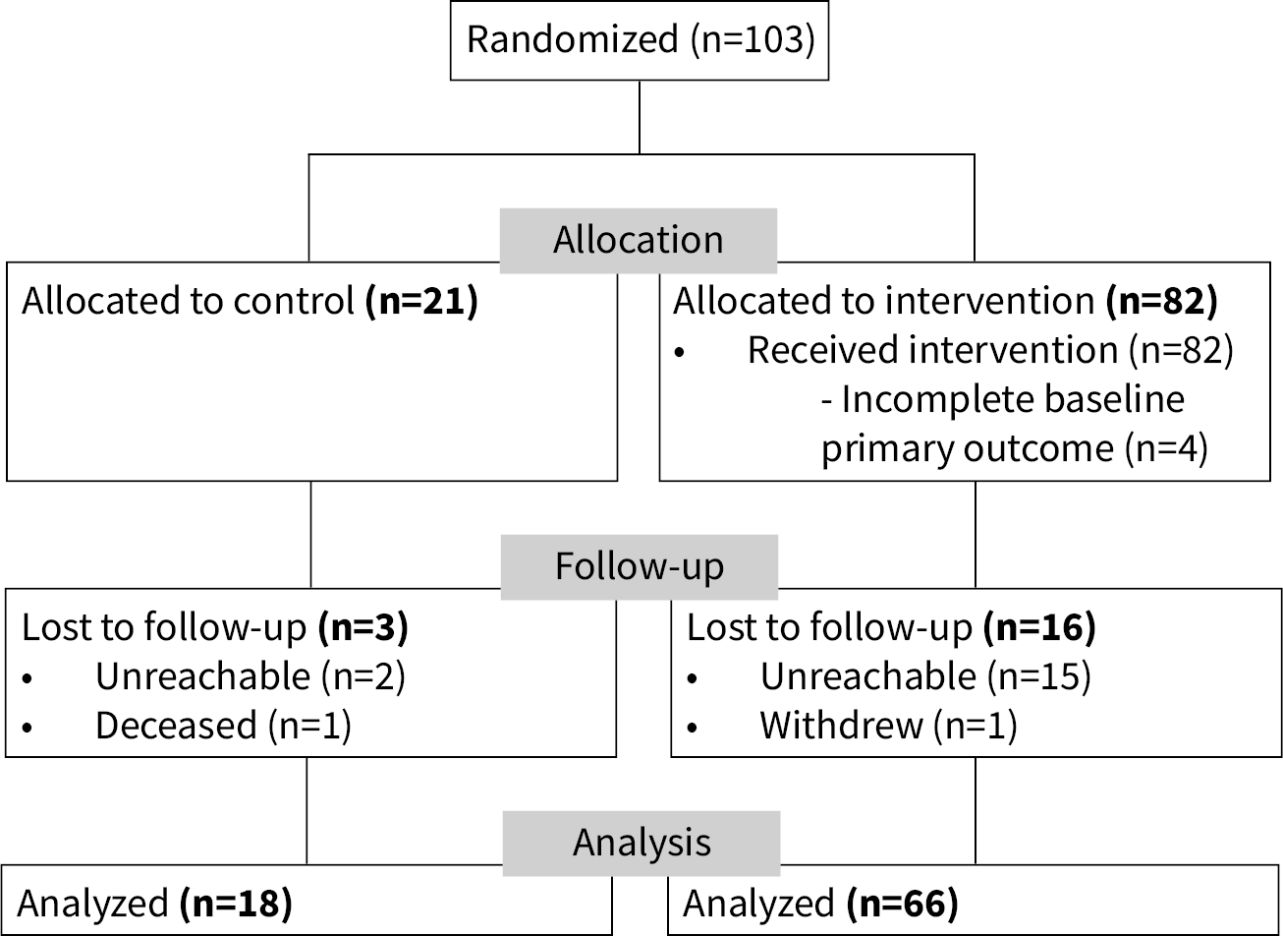
Flowchart of participants included in the CHAT-AF randomized controlled trial. CHAT-AF: Coordinating Health Care With Artificial Intelligence–Supported Technology for Patients With Atrial Fibrillation.

Between January and July 2021, we enrolled 103 participants (82 intervention and 21 control; Figure 1). We lost 16 intervention participants and 3 controls to follow-up (18.4%), and the primary outcome analysis included 66 intervention participants and 19 controls. The follow-up period was from July 2021 to April 2022. Mean age was 63.7 (SD 11.2) years and 69.9% were males (Table 1). The majority were nonuniversity graduates (75.5%), of non-Caucasian ethnicity (24.5%), and had paroxysmal AF (76.7%). Detailed characteristics are provided online (Multimedia Appendix 1).

**Table 1.** Participant baseline characteristics (N=103).

		Control (n=21)	Intervention (n=82)	Total (n=103)
Age^a^ (years), mean (SD)		63.0 (12.1)	63.8 (11.0)	63.7 (11.2)
BMI^a^ (kg/m^3^), mean (SD)		30.9 (5.1)	31.6 (6.8)	31.4 (6.5)
Sex^a^, n (%)				
	Male	14 (66.7)	58 (70.7)	72 (69.9)
	Female	7 (33.3)	24 (29.3)	31 (30.1)
Blood pressure^a^ (mm Hg), mean (SD)				
	Systolic	130.7 (17.2)	132.1 (18.0)	131.8 (17.8)
	Diastolic	78.8 (14.6)	77.6 (13.4)	77.8 (13.6)
Ethnicity, n (%)				
	Non-Caucasian	4 (19)	21 (25.9)	25 (24.5)
Education, n (%)				
	Nonuniversity graduate	13 (61.9)	64 (79)	77 (75.5)
Annual household income in AUS $^b^, n (%)				
	0-31,199	4 (28.6)	9 (16.7)	13 (19.1)
	31,200-77,999	3 (21.4)	20 (37)	23 (33.8)
	78,000+	7 (50)	25 (46.3)	32 (47.1)
Smoking status, n (%)				
	Never smoked	8 (38.1)	39 (50)	47 (47.5)
	Current smoker	0 (0)	7 (9)	7 (7.1)
	Ex-smoker	13 (61.9)	32 (41)	45 (45.5)
Type of AF^a^^c^ (most recent), n (%)				
	Paroxysmal	15 (71.4)	64 (78)	79 (76.7)
	Persistent	5 (23.8)	17 (20.7)	22 (21.4)
	Permanent	1 (4.8)	0 (0)	1 (1)
	Unspecified	0 (0)	1 (1.2)	1 (1)
Time since initial AF diagnosis (years), n (%)				
	<5	13 (61.9)	44 (56.4)	57 (57.6)
	≥5	8 (38.1)	34 (43.6)	42 (42.4)
Medical history^a^, n (%)				
	Hypertension	12 (57.1)	54 (65.9)	66 (64.1)
	Hyperlipidemia	10 (47.6)	39 (47.6)	49 (47.6)
	Heart failure	7 (33.3)	23 (28)	30 (29.1)
	Vascular disease	8 (38.1)	24 (29.3)	32 (31.1)
	Stroke	2 (9.5)	6 (7.3)	8 (7.8)
	Diabetes type 2	5 (23.8)	15 (18.3)	20 (19.4)
	Obstructive sleep apnea	6 (28.6)	33 (40.2)	39 (37.9)
CHA_2_DS_2_-VASC^d^ score, mean (SD)		2.7 (2.2)	2.5 (1.6)	2.6 (1.7)
Medications^a^, n (%)				
	Antiarrhythmic	14 (66.7)	63 (76.8)	77 (74.8)
	Anticoagulation	15 (71.4)	68 (82.9)	83 (80.6)
	Statin	12 (57.1)	46 (56.1)	58 (56.3)
	Angiotensin-converting enzyme inhibitor	3 (14.3)	16 (19.5)	19 (18.4)
	Angiotensin receptor blocker	4 (19)	28 (34.1)	32 (31.1)
	Calcium channel blocker	5 (23.8)	13 (15.9)	18 (17.5)
	Neprilysin inhibitor	1 (4.8)	7 (8.5)	8 (7.8)

a Information collected by clinical investigators from the electronic medical record.

b The average conversion rate during the study was AUS $1=US $0.73.

c AF: atrial fibrillation.

d CHA_2_DS_2_-VASC: congestive heart failure, hypertension, age 75 years and older (doubled), diabetes, stroke (doubled), vascular disease, aged 65 to 74 years, and sex category (female).

### Primary and Secondary Outcomes

**Figure 2.**
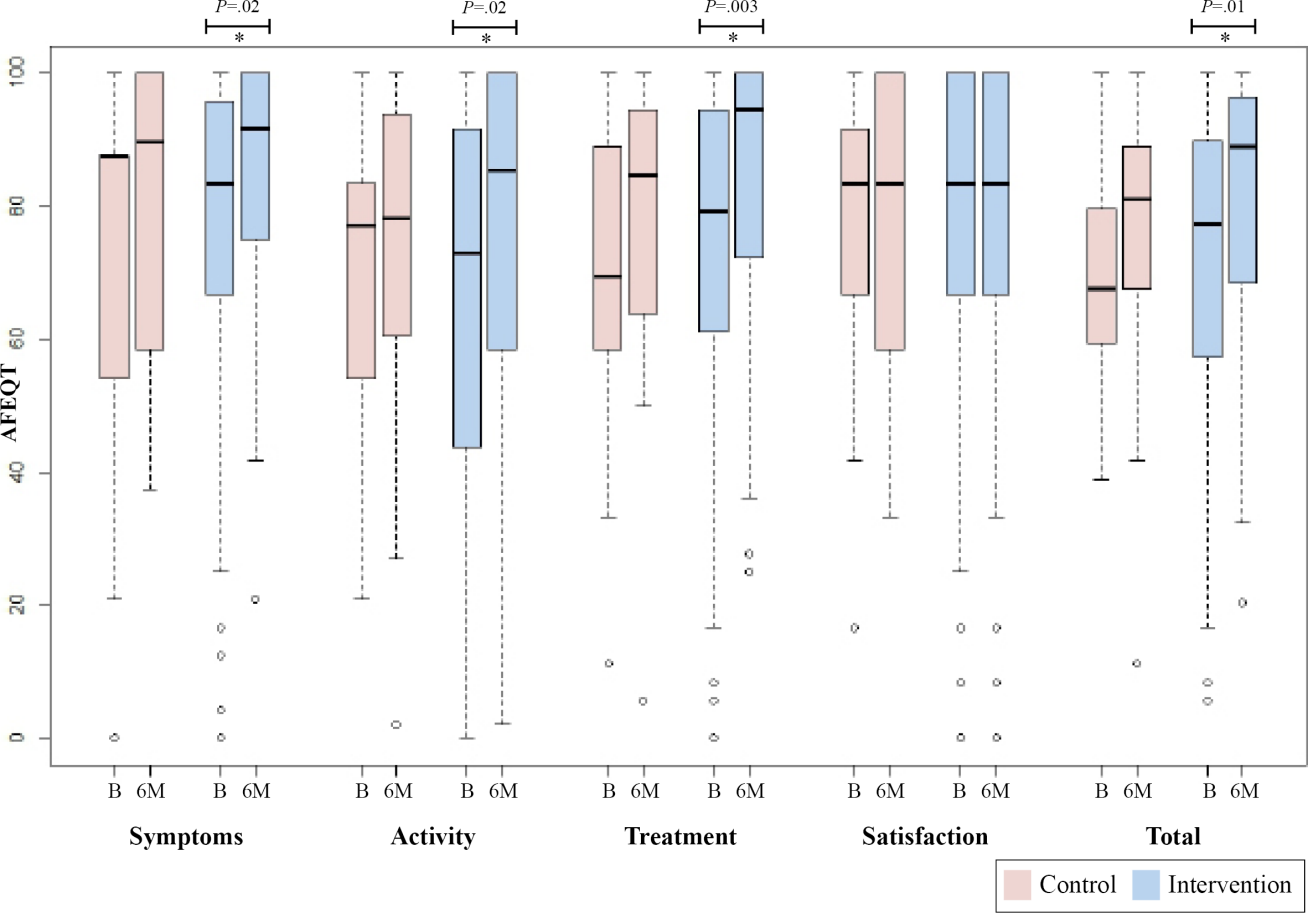
Box plot of AFEQT total and subscale scores. AFEQT scores for intervention (n=66) and control (n=18) groups at B and 6M. Total AFEQT score is an average of symptoms, activity, and treatment subscales (does not include satisfaction). * Significant difference of prepost Wilcoxon 2-tailed *t* test. AFEQT: Atrial Fibrillation Effect on Quality-of-Life; B: baseline; 6M: 6 months.

No significant difference was observed between groups in the primary outcome of AFEQT total score (2.08, 95% CI −7.79 to 11.96; *P*=.46; Figure 2 and Table 2). There were 18.4% missing primary outcome data at 6 months; however, we found no evidence for differences in missingness based on age (grouped by 65 y or older), gender, or ethnicity (grouped by Caucasian or non-Caucasian). Prespecified sensitivity analyses were conducted; both imputation of 3-month AFEQT total score carried forward and baseline imputation revealed similar results to the primary analysis.

**Table 2.** Primary outcome—AFEQT^a^.

AFEQT	Control			Intervention				
	Baseline, mean (SD; n=21)	Follow-up, mean (SD; n=18)	Change, mean (95% CI)	Baseline, mean (SD; n=78)	Follow-up, mean (SD; n=66)	Change, mean (95% CI)	Difference, mean (95% CI)	*P* value^b^
Total score	70.3 (17.0)	74.9 (22.7)	5.0 (−6.1 to 6.0)	69.9 (25.0)	79.9 (20.5)	7.1 (2.9 to 11.3)	2.08 (−7.79 to 11.96)	.46
Symptom	72.0 (29.1)	79.9 (20.9)	6.9 (−9.8 to 23.6)	74.3 (26.8)	83.1 (19.2)	5.8 (0.5 to 11.1)	−1.14 (−14.43 to 12.15)	.69
Activity	69.4 (21.9)	70.6 (27.2)	1.3 (−9.1 to 11.6)	65.6 (30.6)	75.0 (26.6)	7.2 (1.3 to 13.1)	5.92 (−6.59 to 18.44)	.35
Treatment	70.2 (24.4)	77.5 (24.5)	8.6 (−5.7 to 23.0)	72.7 (26.0)	84.3 (20.3)	7.7 (3.5 to 12.0)	−0.90 (−11.85 to 10.05)	.54
Satisfaction	76.6 (21.8)	76.9 (24.0)	1.4 (−13.9 to 16.7)	76.7 (25.6)	80.1 (24.1)	2.3 (−4.3 to 8.9)	0.88 (−14.05 to 15.82)	.69

a AFEQT: Atrial Fibrillation Effect on Quality-of-Life.

b Adjusted analysis consisted of an analysis of covariance test, adjusting for baseline level to estimate the difference between groups at 6 months. Atrial Fibrillation Effect on Quality-of-Life questionnaire scores range from 0‐100 (higher scores associated with better health-related quality of life). The total Atrial Fibrillation Effect on Quality-of-Life score is an average of all subscales (total score, symptom, activity, and treatment), excluding satisfaction.

No difference was observed between groups in the change in AFEQT subscale scores (Figure 2, Table 2). Additional exploratory analyses revealed an improvement in AFEQT total score postintervention in the intervention group (baseline: 69.9, 95% CI 64.4 to 75.5; 6-months: 79.9, 95% CI 74.9 to 84.8; *P*=.01), with no improvements in the control group (Figure 2). Within-group differences revealed the intervention group improved in most AFEQT subscales postintervention (symptom, daily activity, treatment, *P* values<.05), with no improvements seen in the control group (Figure 2, Table 2). No significant differences were observed in AFEQT total and subscale scores from baseline to 3 months between groups (Multimedia Appendix 1).

No significant differences were observed in secondary outcomes of knowledge (AF knowledge), patient activation (Patient Activation Measure), patient assessment of care quality and self-management (Patient Assessment of Chronic Illness Care), or lifestyle behaviors (exercise, fruit and vegetable intake, alcohol intake, and smoking) at 6 months (Multimedia Appendix 1). No significant difference was observed between groups in the proportion that were adherent to medications, visited a GP or cardiologist, visited the emergency department, or were hospitalized and had an AF procedure (Multimedia Appendix 1). A total of 3% of intervention participants had a stroke or myocardial infarct in the previous 6 months compared to no controls (Multimedia Appendix 1).

### Process Evaluation

**Figure 3.**
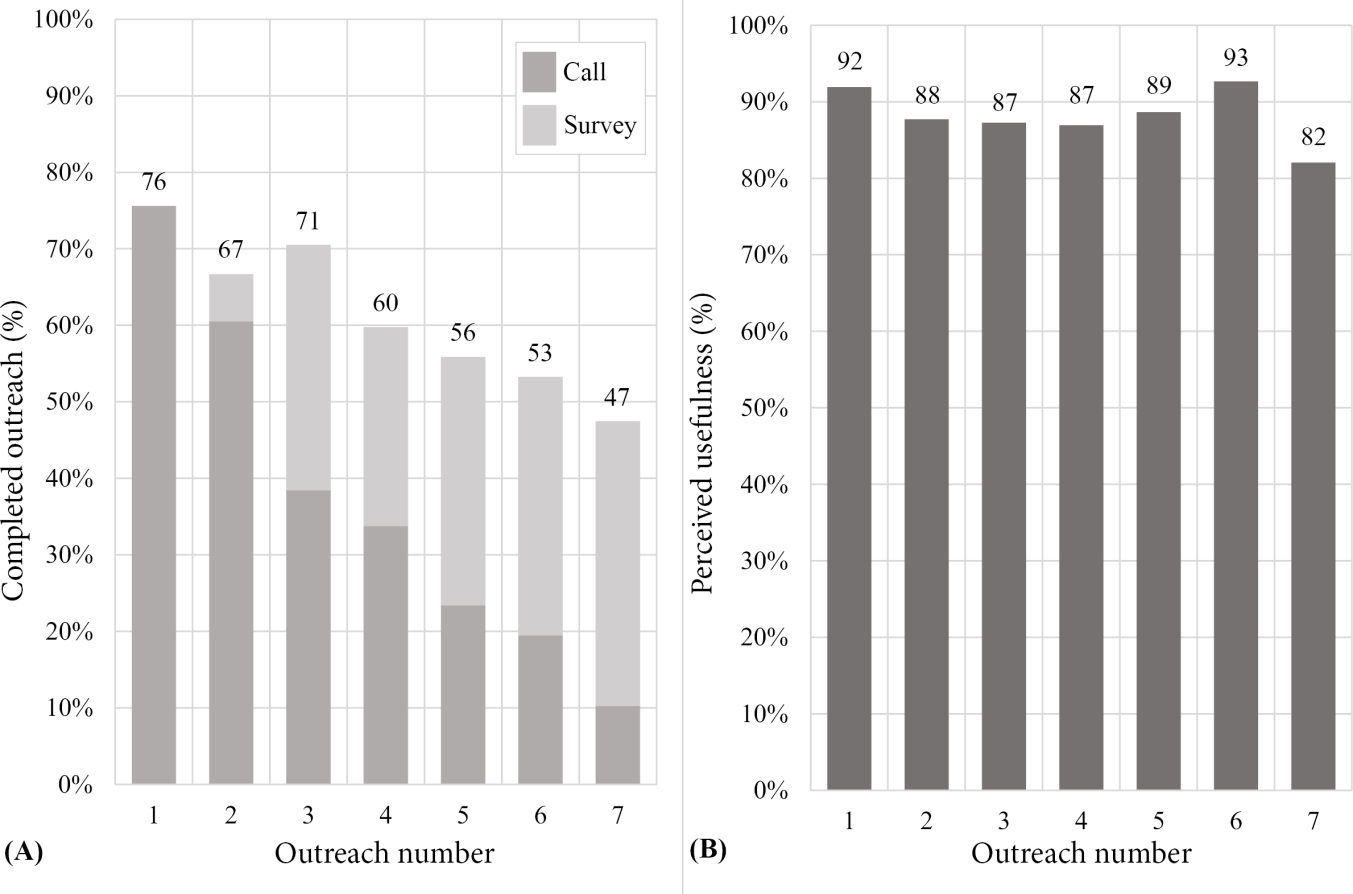
CHAT-AF intervention (A) outreaches completion and (B) perceived usefulness. Outreaches 1 to 7 occurred at 24‐48 hours, 2 weeks, 1 month, 2 months, 3 months, 4 months, and 5 months posthospital discharge, respectively. CHAT-AF: Coordinating Health Care With Artificial Intelligence–Supported Technology for Patients With Atrial Fibrillation.

A total of 338 of 550 outreaches were delivered via the conversational AI calls, and of these, 226 (66.9%) were completed (original delivery; Multimedia Appendix 1). The remaining 212 outreaches were delivered only by a survey tool, and of these, 112 (52.8%) were completed (modified delivery). The completion rate of the first outreach was 75.6% (62 calls and 0 surveys), and this dropped to 47.4% (8 calls and 29 surveys) by the final outreach, with an average completion rate across the 7 outreaches of 61.5% (Figure 3). On average, each participant completed 4.12 of 7 outreaches. A total of 51 participants (62.2%) had higher engagement (completed 4 or more outreaches), and there were no demographic variables influencing this outcome (age, gender, ethnicity, education, type of AF, time since AF diagnosis, and CHA_2_DS_2_-VASC). Most participants (56.1%) visited the educational website at least once, and the mean number of visits was 5.54 times. The most visited topic was general information about AF (138 visits), which included videos narrated by a local cardiologist. In terms of perceived usefulness, 88.4% of completed outreach was reported as useful (Figure 3) and this was similar in the original (89.1%) and the modified delivery (87.6%). Detailed process evaluation results are provided online (Multimedia Appendix 1).

## Discussion

### Principal Results

The CHAT-AF study provides a proof-of-concept with initial data on the efficacy of a novel digital health follow-up strategy for patients with AF that leverages conversational AI. On average, participants completed 4 of 7 outreaches, and 88.4% of completed outreaches were reported as useful, suggesting this approach to delivering care is feasible and could enable chronic disease services to follow up with patients at scale with fewer in-person and staffed visits. Due to challenges with the technology partner acquisition, this study had only recruited 27% of its intended sample size and was subsequently underpowered for its primary outcome. The trial did not demonstrate a significant difference in the primary outcome between intervention and control; however, there was a benefit suggested by an exploratory prepost comparison that showed overall AF-QoL improved at 6 months in the intervention group from baseline, with no similar improvement observed in the control group.

### Comparison With Prior Work

Digital support programs show promise in improving QoL for patients with AF, but more robust studies are needed to determine effectiveness. Despite this study’s limited power to determine efficacy, other trials have reported improvements in AF-QoL, knowledge, medication adherence, and clinical outcomes, with similar digital health interventions (without conversational AI) that delivered a combination of health education, monitoring, and self-management support for patients with AF [4-6,25]. However, the paucity of literature in this space has been confined to mobile apps [4,6,25-27], text messaging [26,28], and web-based platforms [5,8,9], many of which have reported limited engagement. The current intervention was unique in its approach to interactively engage with patients through conversational technology. A comparable study by Guhl et al 2020 [4] involved an embodied conversational agent that consisted of a virtual avatar displaying verbal and nonverbal gestures to deliver education and heart rhythm monitoring support to patients with AF [4]. Participants interacted with the conversational agent 18 times over 30 days and had improved medication adherence and AF-QoL compared with usual care [4]. Notably, this intervention did not include an AI component and required users to respond to queries by clicking on prespecified options in the mobile app, rather than using speech recognition technology, which was used in CHAT-AF to facilitate more natural dialogue and better engagement.

AI-enabled conversational technologies allow for more human-like interactions and have shown promise in other populations. Existing trials of conversational AI have demonstrated improvements in insulin adherence and glycemic control in patients with type 2 diabetes [12], medication adherence in patients with hypertension and diabetes [11], and symptoms of depression and anxiety in college students [13,14]. These studies reported good engagement over a 2-week period, where college students exchanged 283 messages with a chatbot [14], and undertook 12 check-ins with another chatbot [13] regarding their mental health. Another study that used a voice-based conversational AI interface, resembling the phone calls in the current intervention, found patients with type 2 diabetes logged daily insulin use and fasting blood glucose levels almost every day for about 4 months [12]. Similar to the phone call–based delivery mode used in the current study, another study delivered cognitive behavioral therapy for pain management using voice-based conversational-AI phone calls and reported that 87.8% of calls were completed [29]. In our study, the transition from phone call to solely survey-based delivery may have resulted in completion rates (67%) lower than reported in the aforementioned study. Overall, evidence suggests conversational AI technologies can successfully engage and support patients with chronic disease management, but additional studies are needed to evaluate long-term engagement.

The deeper level of human-technology interaction allowed by conversational AI seems a key factor in achieving higher user engagement [12-14,30-32]. The unplanned change in intervention delivery in the current study offered an important opportunity to compare engagement between different communication modes. Interestingly, the shift from conversational AI phone calls to surveys resulted in a drop in overall outreach completion rates (from 66.9% to 52.8%). A hypothesized explanation may be the stronger appeal of the human-like interactions provided by these technologies and their ability to facilitate natural dialogues with users. This is in line with existing work indicating the possibility of a relationship between humans and nonhuman agents in the context of health [12-14]. As digital health interventions increasingly incorporate elements that make them interactive, adaptive, persuasive, and personalized, they become more able to reproduce elements of a therapeutic relationship and can better engage and support patients in their health journey [33].

The early stopping of the trial due to suspended delivery of the intervention by the technology partner posed challenges that are important to consider. For the trial steering committee, it was key that enrolled participants received the entirety of the 6-month program, to satisfy the duty of care for enrolled patients as well as to ensure fidelity in delivery of all educational content in the program. The decision to use REDCap [16] surveys when the automated phone calls had to be stopped offered a satisfactory solution to these concerns and also provided the opportunity to observe feasibility measures such as engagement and perceived usefulness rates between these 2 modes of delivery while the other elements were unchanged [30,31]. An attenuating factor for early stopping was that the trial steering committee was forewarned of when the company was going to withdraw services, and that allowed for adequate time to develop an alternative solution and streamline the transition for participants. Other articles have reported early trial stopping due to technology partner withdrawal of services [34]. Open communication and goal alignment have been proposed as key to achieving solutions that can benefit all parties involved and bridge the gap in “academia-industry” relationships [35,36]. Stronger partnership between sectors is increasingly needed as collaboration between technological, research, and clinical expertise is paramount to successfully implement digital health solutions.

### Implications

The growing prevalence of cardiovascular diseases such as AF, which require ongoing management to prevent frequent hospitalization, puts a significant toll on health care resources. Interventions such as CHAT-AF have the potential to provide support at scale, with a risk management system that allows patients at risk of deteriorating to be identified, prioritized, and managed appropriately. Using phone calls to provide patient support has advantages over other mobile technologies (eg, apps and wearable devices) as it is not dependent on internet connectivity, phone models, or operating systems. Digital interventions such as CHAT-AF could be used to provide support to patients with AF in the community, including remote and rural areas where patients have traditionally had poorer access to health care services [37]. As demonstrated in this proof-of-concept study, the engagement, perceived usefulness, and initial suggestive findings of AF-QoL improvement argue that this technology should be further examined in larger RCTs.

### Limitations

This study has several limitations that need to be considered. This study was limited in not achieving its target sample size and multicenter reach due to premature study completion, and therefore, careful consideration needs to be made when interpreting the results as this study was underpowered. This also meant some participants on the intervention arm were unable to complete their conversational AI “visits” later into the planned follow-up period, which is likely to have contributed to the ability to evaluate the effectiveness of the intervention on outcomes. The authors are in the process of conducting a large multicenter RCT evaluation of an optimized version of the current intervention, to address the power, sample size, and interpretation limitations of this study (registered with the Australian and New Zealand Clinical Trials Registry, ACTRN12623000850673). Moreover, except for baseline characteristics (medical history), all outcome data were self-reported. There was a difference in attrition between the intervention (16/82) and control (3/21) groups, which may have impacted the primary analysis, and while there was no evidence of bias for missingness, caution needs to be taken in interpretation due to the limitations in lack of power and changes in intervention delivery. This study’s population was younger (mean age of 63.7 y) than the average patients with AF population in Australia (mean age of 75 y) [38], which may have occurred due to the higher likelihood of younger patients’ comfort and interest in participating in technology-based research [35]. Perceived usefulness was assessed as the final question of each outreach and therefore results are based on individuals that completed the particular outreach and its final question—this may not be reflective of overall intervention usefulness, as it does not reflect outreaches not completed by participants (344 of 550 outreaches were completed by participants and were rated in terms of their perceived usefulness, of these, 304 of the 344, 88.4%, were assessed positively based on usefulness). However, to capture the perspectives of a diverse group of the intervention cohort, we conducted qualitative interviews using purposive sampling techniques to ensure we captured the experiences of those with varying levels of engagement [38,39]. Furthermore, the study was delivered in English and included predominately Caucasian participants, highlighting the need for further work to validate this technology in larger trials with more diverse and representative patient populations.

### Conclusions

This study found that a conversational AI follow-up program for patients with AF improved AF-QoL postintervention (but not compared with usual care). As the burden of AF continues to grow, novel technologies that can interact with patients and support them in their care journey will be needed, and digital health can provide this at a scalable level. However, larger-scale RCTs and implementation studies are needed to determine the effectiveness of conversational AI in improving AF outcomes.

## Supplementary material

10.2196/64326 Multimedia Appendix 1 Intervention details, outcome measures, and detailed analyses.

10.2196/64326 Multimedia Appendix 2 Statistical analysis plan.

10.2196/64326 Checklist 1 CONSORT-EHEALTH checklist. CONSORT: Consolidated Standards of Reporting Trials.

## Abbreviations

AF: atrial fibrillation
AF-QoL: Atrial Fibrillation Specific Quality of Life
AFEQT: Atrial Fibrillation Effect on Quality-of-Life
AI: artificial intelligence
CHA_2_DS_2_-VASC: congestive heart failure, hypertension, age 75 years and older (doubled), diabetes, stroke (doubled), vascular disease, aged 65 to 74 years, and sex category (female)
CHAT-AF: Coordinating Health Care With Artificial Intelligence–Supported Technology for Patients With Atrial Fibrillation
CONSERVE: CONSORT (Consolidated Standards of Reporting Trials) and SPIRIT (Standard Protocol Items: Recommendations for Interventional Trials) Extension for RCTs (Randomized Controlled Trials) Revised in Extenuating Circumstances
CONSORT: Consolidated Standards of Reporting Trials
GP: general practitioner
HMS: Health Management Systems, Inc
QoL: Quality of Life
RCT: randomized controlled trial
REDCap: Research Electronic Data Capture
SPIRIT: Standard Protocol Items: Recommendations for Interventional Trials
