# Supplementary material for: Conversational AI Phone Calls to Support Patients With Atrial Fibrillation: Randomized Controlled Trial
Source: JMIR Cardio. 2025 Aug 19;9:e64326. doi: 10.2196/64326 (PMC12364416; doi:10.2196/64326)
Supplement: Multimedia Appendix 1 [file cardio-v9-e64326-s001.pdf]

Contents

Figure S1. CHAT-AF intervention **2**

Figure S2. CHAT-AF education website screenshots **3**

Figure S3. CHAT-AF survey outreach interface screenshots **3**

Table S1. Trial outcomes and baseline characteristics **4**

Table S2. Detailed participant characteristics of CHAT-AF study, including 21 controls and 82 intervention participants **6**

Table S3. AFEQT at baseline and 3 months in control and intervention groups **7**

Table S4. Secondary outcomes of knowledge, patient activation, patients' assessment of care and self-management support and lifestyle behaviour outcomes **7**

Table S5. Secondary outcomes of medication adherence, healthcare service utilisation patterns, health outcomes **8**

Table S6. Outreach completion for n=82 intervention group participants, where completed is indicative of ≥50% of questions are answered in the outreach **9**

Table S7. Engagement defined as the number of completed outreaches for n=82 intervention group participants, where completed is indicative of ≥50% of questions are answered in the outreach **9**

Table S8. Univariate logistic regression models predicting the binary outcome (YES/NO) of higher individual engagement (≥4 outreaches completed) **9**

Table S9. Interactions with the educational website for n=82 intervention group participants **10**

Table S10. Outreach perceived usefulness for n=82 intervention group participants, proportion of participants that replied 'yes' to the question, "Did you find the information in this outreach helpful?" divided by participants that attempted the outreach (answered ≥1 question) **10**

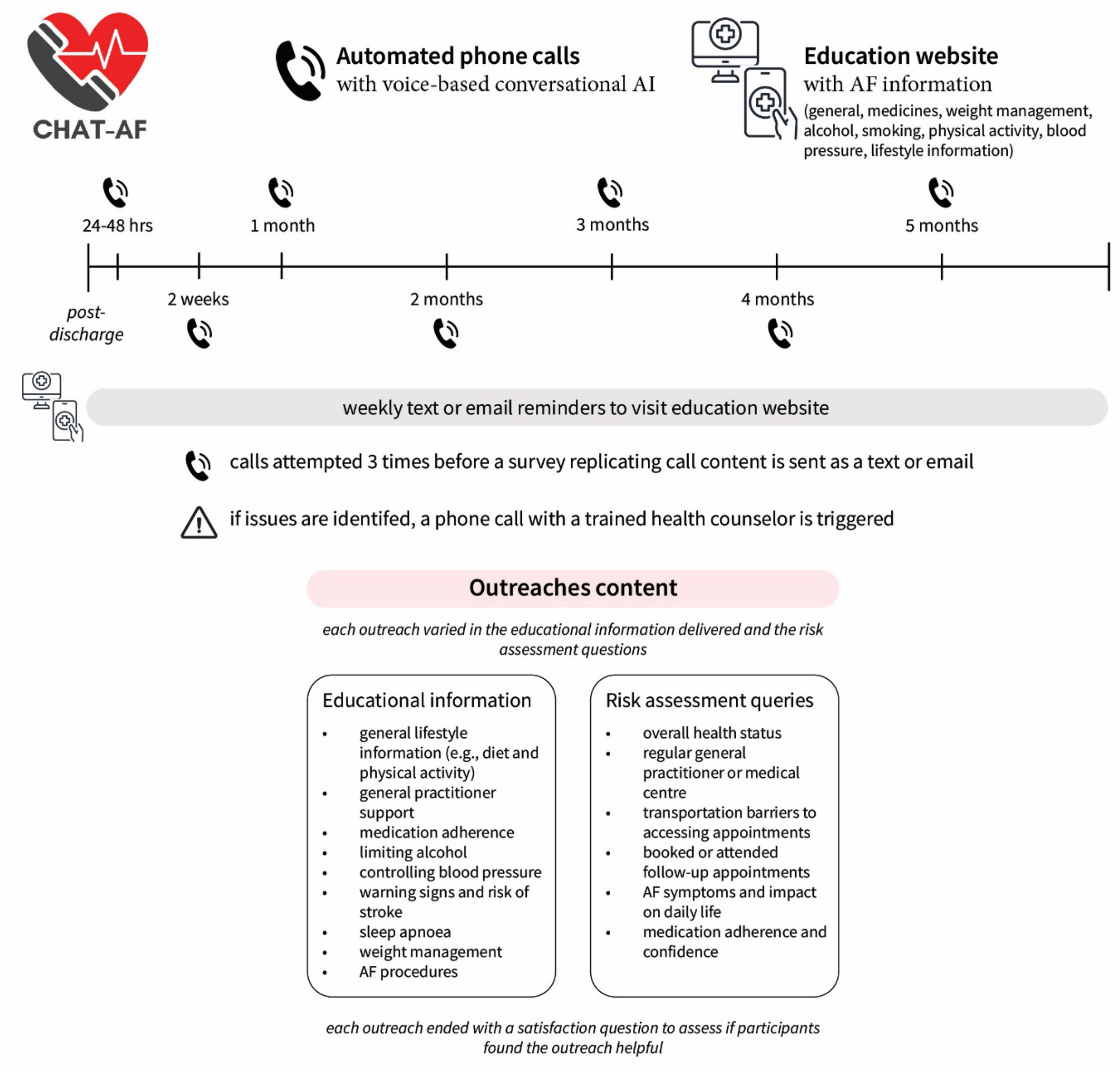

Figure S1. CHAT-AF intervention

Abbreviations: AF, atrial fibrillation; AI, artificial intelligence. The intervention was modified after the technology partner withdrew and the call content was delivered only via the survey tool. The education website was also redeveloped to match the original.

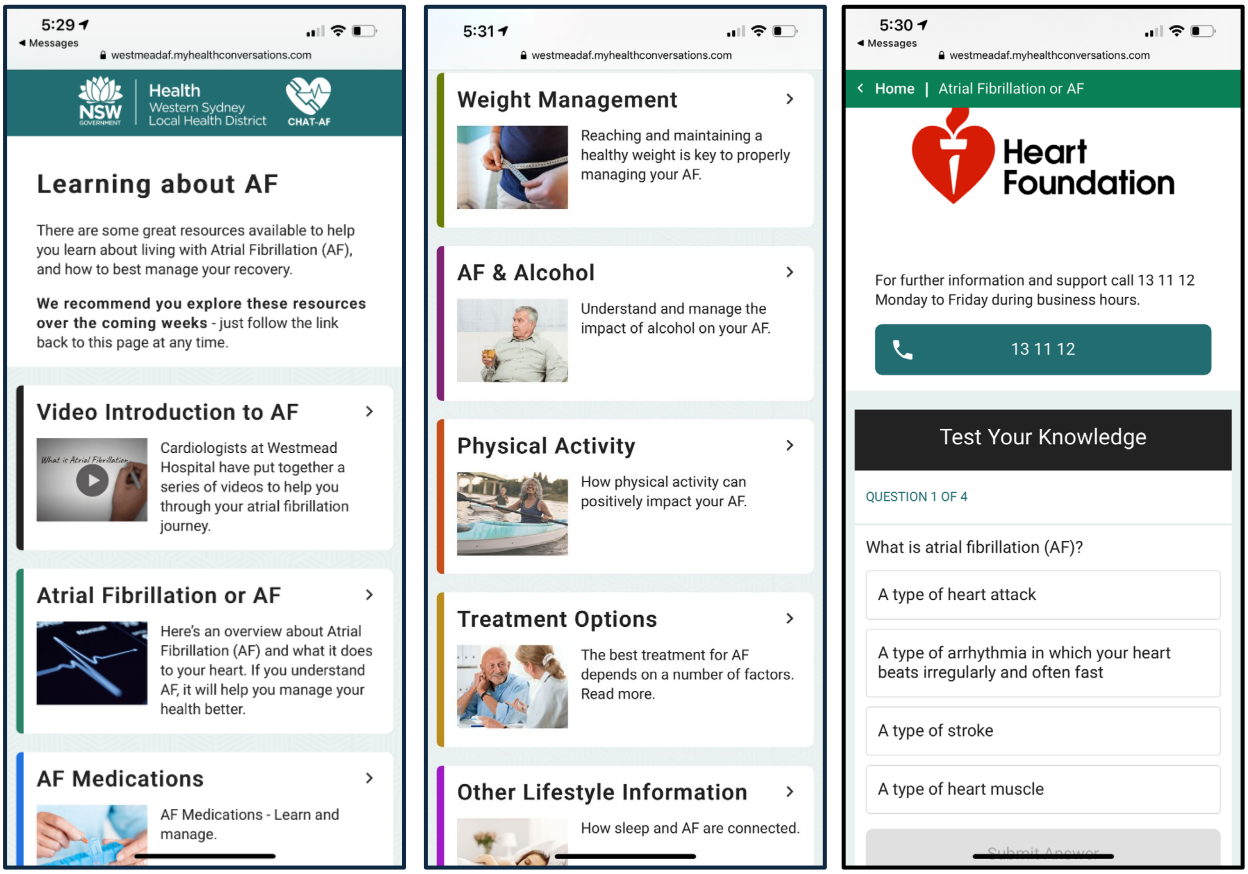

Figure S2. CHAT-AF education website

screenshots. Abbreviations: AF, atrial fibrillation.

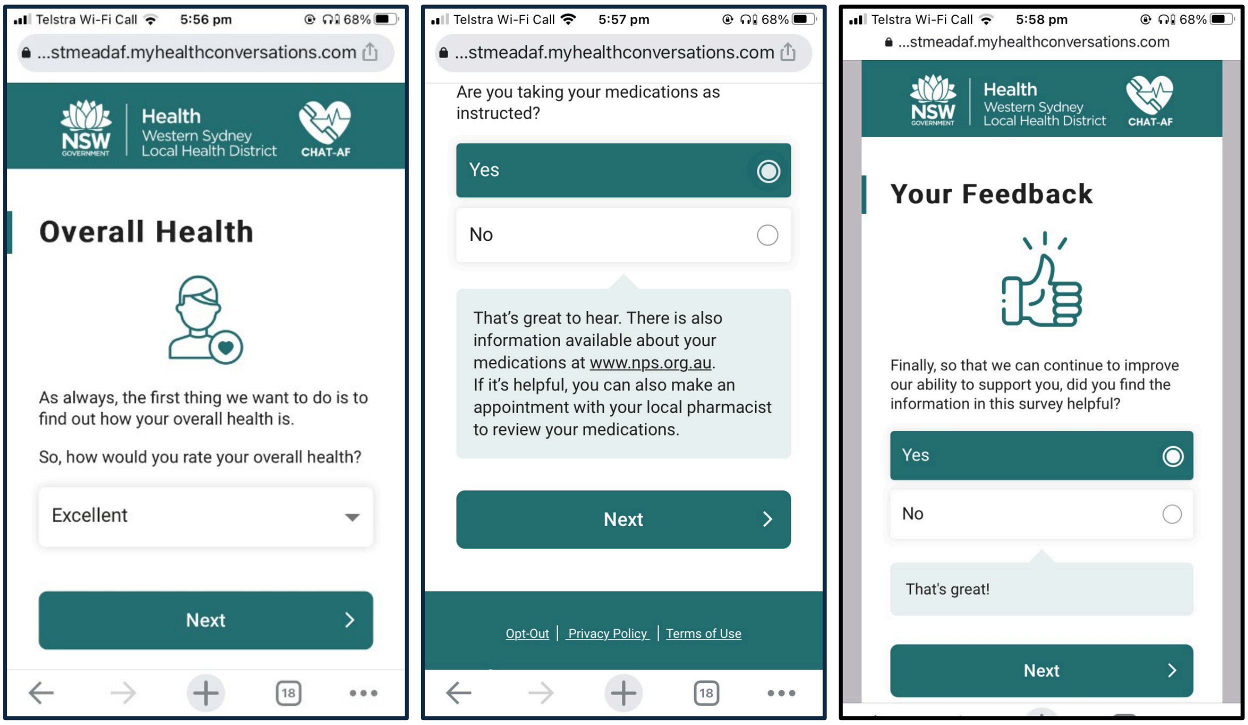

Figure S3. CHAT-AF survey outreaches interface screenshots.

Table S1. Trial outcomes and baseline characteristics

Outcome/ Data	Collection	Time	Definition
Demographics	Self-reported	B	Sex, ethnicity, education, household income, smoking status
Anthropometrics and medical history	Medical records	B	Date of birth (used to calculate age), height, weight, blood pressure (systolic and diastolic), past medical conditions, medications, AF diagnosis year, type of AF
Quality of Life	Self-reported	B, 3M, 6M	The Atrial Fibrillation Effect on QualiTy-of-life (AFEQT) is a 20-item (7-point Likert scale) validated questionnaire for assessing AF related QoL, and it outputs a total score and four sub-scales scores (symptom, daily activity, treatment and satisfaction) all ranging from 0-100 (higher scores associated with better health-related-QoL). Total AFEQT is an average of all subscales, excluding satisfaction. The primary outcome was change in AFEQT total from baseline to 6 months
Knowledge	Self-reported	B, 3M, 6M	The AF Knowledge Scale is a validated questionnaire was used to assess patients' knowledge of AF. It consists of 10 assessment questions and the total score is representative of the number of correct responses. For the current study, the last item (11th question) was removed given that it referred to the 'thrombosis center' which does not exist in Australia.
Patient activation	Self-reported	B, 6M	The Patient Activation Measure (PAM)-13 is a validated questionnaire that evaluates the patient's perceived knowledge, skills, and confidence in self-management activities. Question responses are structured according to a Likert scale from 1 (strongly disagree) to 4 (strongly agree) and total scores range from 0 to 100 (based on provided calibration tables), where higher scores represent more patient activation in disease self-management.
Patient's assessment of care quality and self-management support	Self-reported	B, 6M	The Patient Assessment of Chronic Illness Care (PACIC) is a 20-item (5-point Likert scale) validated questionnaire that consists of five subscales (patient activation, decision support, goal setting, problem solving and follow-up) and an overall summary score (average of completed items) ranging from 0 to 5, where higher scores are reflective of better patient-health care team interactions and self-management support.
Exercise	Self-reported	B, 6M	Exercise minutes per week – "In a typical week, how much time do you spend exercising? (This includes brisk walking, swimming, yoga, skipping, cycling, sporting activities)"<br>Self-reported measures >840 minutes per week were replaced with equal to 840 minutes (for data cleaning purposes) and categorised as meeting the ≥ 150 minutes METs guideline recommendations for weekly exercise.
Alcohol	Self-reported	B, 6M	Alcoholic drinks consumed per week – "How many standard drinks of alcohol do you drink on average per week?"
Fruit and vegetable intake	Self-reported	B, 6M	Daily fruit and vegetable intake – "How many serves of fruit/vegetables do you usually eat per day?"<br>(one serve = medium piece or two small pieces of fruit or one cup of diced pieces)<br>(one serve = 1/2 cup cooked vegetables or 1 cup of salad vegetables)
Cigarette smoking	Self-reported	B, 6M	Daily cigarette smoking – "How many cigarettes do you smoke per day?"
Medication adherence	Self-reported	B, 6M	Missed medication days in past week – "In the last 7 days, on how many days did you miss a dose of any of your prescribed medications?" Definition: Yes = 0 = adherent; No = 1-7 = non-adherent
General practitioner /cardiologist visit	Self-reported	B, 6M	GP or cardiologist visit in previous 6 months<br>• "In the past 6 months, how many times have you seen your GP because of heart problems or heart symptoms? Please note this includes face-to-face and telephone appointments."

			• "In the past 6 months, how many times have you seen a heart specialist (cardiologist)? Please include today's appointment and all telephone appointments."<br>If the answer to either of the above questions is ≥ 1 number of times then it is YES, or if < 1 number of times then it is NO
Emergency department presentation/ hospitalisation	Self-reported	B, 6M	Emergency department presentation or hospitalisation in previous 6 months<br>• "In the past 6 months, how many times have you visited the emergency department (ED) because of possible heart symptoms or problems".<br>• "In the past 6 months, how many times have you been hospitalised because of heart problems (e.g. atrial fibrillation, stroke, heart attack or heart failure)?"<br>If the answer to either of the above questions is ≥ 1 number of times then it is YES, or if < 1 number of times then it is NO
Ablation/cardioversion procedure	Self-reported	B, 6M	Ablation or cardioversion procedure in previous 6 months<br>• "In the past 6 months, have you had a catheter ablation procedure to treat your atrial fibrillation? Please note a catheter ablation is a procedure that finds where your abnormal heart rhythm comes from and treats that area of the heart."<br>• "In the past 6 months, have you had a cardioversion procedure to treat your atrial fibrillation? Please note a cardioversion is a procedure that uses an electrical current to reset your heart rhythm."<br>Definition: If the answer to either of the above questions is YES
Health outcomes: stroke/ myocardial infarct	Self-reported	B, 6M	Stroke or heart attack in the previous 6 months – "In the past 6-months have you had a stroke?" and "In the past 6-months have you had a heart attack?" Definition: If the answer to either of the above questions is YES
**Process evaluation outcomes (intervention group only)**			
Outreach completion	Intervention metrics	6M	Completion rate of each outreach was calculated by successful completion (≥ 50% of the questions answered in the calls/surveys), divided by the number of participants that received the outreach. This was done for all outreaches 1 to 7.
Outreach perceived usefulness	Intervention metrics	6M	Usefulness for each outreach was defined as the number of individuals that answered, 'Yes' to the question 'Did you find the information in this call/survey helpful?' and was divided by the number of participants that attempted the outreach (≥1 question answered). This was done for all outreaches 1 to 7.
Individual engagement (calls/surveys)	Intervention metrics	6M	Individual engagement was the total number of outreaches (calls/surveys) completed (defined as ≥ 50% of the questions answered in the calls/surveys) by the participant
Individual engagement (website)	Intervention metrics	6M	Mean number of visits on the education website for each participant.
Abbreviations: B, baseline; 3M; 3 months; 6M, 6 months; AF, atrial fibrillation; AFEQT, Atrial Fibrillation Effect on QualiTy-of-life; QoL, Quality of Life, PAM-13, Patient Activation Measure (PAM)-13; PACIC, Patient Assessment of Chronic Illness Care; MET, metabolic equivalent of task; GP, general practitioner; ED, emergency department.			

Table S2. Detailed baseline participant characteristics

	Control	Intervention	Total
**Age, years**	**N = 21**	**N = 82**	**N = 103**
Mean (SD)	63.0 (±12.1)	63.8 (±11.0)	63.7 (±11.2)
<65	11 (52.4%)	39 (47.6%)	50 (48.5%)
65-74	6 (28.6%)	30 (36.6%)	36 (35.0%)
≥75	4 (19.0%)	13 (15.9%)	17 (16.5%)
**Body mass index, kg/m^2**	**N = 20**	**N = 73**	**N = 93**
Mean (SD)	30.9 (±5.1)	31.6 (±6.8)	31.4 (±6.5)
underweight	0 (0.0%)	0 (0.0%)	0 (0.0%)
healthy	1 (5.0%)	13 (17.8%)	14 (15.1%)
overweight	7 (35.0%)	22 (30.1%)	29 (31.2%)
obese	12 (60.0%)	38 (52.1%)	50 (53.8%)
**Ethnicity**	**N = 21**	**N = 81**	**N = 102**
Caucasian	17 (81.0%)	60 (74.1%)	77 (75.5%)
Chinese	0 (0.0%)	4 (4.9%)	4 (3.9%)
South Asian (Bangladesh, India, Nepal, Pakistan, Sri Lanka)	0 (0.0%)	2 (2.5%)	2 (2.0%)
Other Asian	0 (0.0%)	4 (4.9%)	4 (3.9%)
Arab or Persian	2 (9.5%)	4 (4.9%)	6 (5.9%)
Other	2 (9.5%)	7 (8.6%)	9 (8.8%)
**Education**	**N = 21**	**N = 81**	**N = 102**
Primary school	0 (0.0%)	2 (2.5%)	2 (2.0%)
Yr 10 School certificate	5 (23.8%)	19 (23.5%)	24 (23.5%)
Yr 12 Higher school certificate	6 (28.6%)	20 (24.7%)	26 (25.5%)
Diploma/Technical	2 (9.5%)	23 (28.4%)	25 (24.5%)
University undergraduate	6 (28.6%)	12 (14.8%)	18 (17.6%)
University postgraduate	2 (9.5%)	5 (6.2%)	7 (6.9%)
**Household income, AUD yearly**	**N = 14**	**N = 54**	**N = 68**
Less than $15,599	1 (7.1%)	5 (9.3%)	6 (8.8%)
$15,600 - $31,199	3 (21.4%)	4 (7.4%)	7 (10.3%)
$31,200 - $51,999	1 (7.1%)	13 (24.1%)	14 (20.6%)
$52,000 - $77,999	2 (14.3%)	7 (13.0%)	9 (13.2%)
$78,000 - $104,000	1 (7.1%)	9 (16.7%)	10 (14.7%)
Over $104,000	6 (42.9%)	16 (29.6%)	22 (32.4%)

Table S3. Atrial Fibrillation Effect on QualiTy-of-life (AFEQT) at baseline and 3 months

	Control			Intervention				
	**Baseline, mean (SD)**	**3 months, mean (SD)**	**Change, mean (95 % CI)**	**Baseline, mean (SD)**	**3 months, mean (SD)**	**Change, mean (95 % CI)**	**Mean difference (95% CI)**	**p-value***
**AFEQT**	n=21	n=16		n=78	n=56			
Total score	70.3 (17.0)	70.1 (24.3)	-2.1 (-14.3 – 10.0)	69.9 (25.0)	75.7 (21.5)	4.6 (-0.3 – 9.4)	6.71 (-4.41 – 17.83)	0.225
Symptom	72.0 (29.1)	78.1 (24.2)	4.2 (-10.9 – 19.2)	74.3 (26.8)	81.6 (17.5)	5.8 (0.9 – 10.8)	1.64 (-10.58 – 13.85)	0.558
Daily activity	69.4 (21.9)	66.3 (30.3)	-4.0 (-21.3 – 13.2)	65.6 (30.6)	69.8 (29.6)	3.9 (-2.8 – 10.7)	7.98 (-7.66 – 23.62)	0.405
Treatment	70.2 (24.4)	70.0 (24.9)	-3.8 (-15.1 – 7.5)	72.7 (26.0)	79.4 (22.3)	5.0 (-0.5 – 10.5)	8.82 (-3.03 – 20.67)	0.085
Satisfaction	76.6 (21.8)	79.2 (23.8)	3.1 (-7.6 – 13.9)	76.7 (25.6)	77.4 (23.4)	0.3 (-6.5 – 7.1)	-2.82 (-16.73 – 11.08)	0.711

AFEQT scores range from 0-100 (higher scores associated with better health-related-QoL). Total score is an average of all subscales, excluding satisfaction. *Adjusted analysis consisted of a ANCOVA test, adjusting for baseline level to estimate the difference between groups at 3 months.

Table S4. Secondary outcomes of knowledge, patient activation, patients' assessment of care and self-management support and lifestyle behaviour outcomes

	Control			Intervention				
**Outcome**	**Baseline, mean (SD)**	**6 months, mean (SD)**	**Change, mean (95% CI)**	**Baseline, mean (SD)**	**6 months, mean (SD)**	**Change, mean (95% CI)**	**Mean difference (95% CI)**	***p-value**
**Knowledge, AF Knowledge Scale**	n=21	n=18		n=76	n=66			
Total score	6.8 (1.7)	6.9 (1.7)	-0.3 (-1.2 – 0.6)	6.3 (1.7)	6.4 (1.4)	0.1 (-0.2, 0.4)	0.35 (-0.41 – 1.12)	0.836
**Patient activation, PAM-13**	n=21	n=18		n=77	n=66			
Level 1 = low activation, n (%)	3 (14.3%)	2 (11.1%)	-	9 (11.7%)	8 (12.1%)	-	-	-
Level 2, n (%)	2 (9.5%)	2 (11.1%)	-	19 (24.7%)	13 (19.7%)	-	-	-
Level 3, n (%)	5 (23.8%)	4 (22.2%)	-	19 (24.7%)	21 (31.8%)	-	-	-
Level 4 = high activation, n (%)	11 (52.4%)	10 (55.6%)	-	30 (39.0%)	24 (36.4%)	-	-	-
Total PAM-13	71.0 (17.3)	74.8 (20.7)	3.0 (-6.1 – 12.1)	64.6 (20.3)	66.7 (21.6)	2.7 (-2.5 – 7.9)	-0.33 (-11.37 – 10.71)	0.423
**Patients' assessment of care and self-management support, PACIC**	n=21	n=18		n=77	n=66			
Patient activation	3.0 (1.0)	3.3 (1.3)	0.0 (-0.8 – 0.7)	3.1 (1.1)	3.0 (1.1)	0.0 (-0.3 – 0.3)	0.0 (-0.69 – 0.69)	0.491
Delivery system design	3.0 (0.9)	3.1 (0.9)	-0.1 (-0.5 – 0.4)	3.3 (0.9)	3.3 (0.8)	0.0 (-0.2 – 0.2)	0.09 (-0.38 – 0.55)	0.339
Goal setting	2.4 (1.1)	2.4 (1.1)	-0.1 (-0.6 – 0.3)	2.7 (0.9)	2.5 (0.9)	-0.1 (-0.3 – 0.1)	0.01 (-0.49 – 0.52)	0.807
Problem solving	3.0 (1.1)	2.8 (1.1)	-0.4 (-0.8 – 0.1)	3.3 (1.2)	3.0 (1.1)	-0.2 (-0.5 – 0.0)	0.11 (-0.46 – 0.68)	0.531
Follow-up	1.9 (0.9)	2.4 (1.2)	0.3 (-0.2 – 0.9)	2.2 (0.9)	2.1 (0.8)	-0.1 (-0.4 – 0.1)	-0.45 (-0.98 – 0.08)	0.115
Total PACIC	2.6 (0.9)	2.7 (1.0)	0.0 (-0.5 – 0.4)	2.8 (0.8)	2.7 (0.8)	-0.1 (-0.3 – 0.1)	-0.08 (-0.51 – 0.35)	0.818
**Lifestyle behaviours**	n=21	n=19		n=78	n=67			

Exercise minutes (weekly)	221.2 (182.1)	255.3 (234.4)	60.8 (-30.0 – 151.7)	202.0 (203.5)	174.6 (163.7)	-30.3 (-75.0 – 14.4)	-91.17 (-188.03 – 5.69)	0.069
>150 minutes METs	13 (61.9%)	13 (68.4%)	-	44 (56.4%)	32 (47.8%)	-	-	-
Fruit servings (daily)	1.8 (0.9)	1.9 (1.1)	0.2 (-0.4 – 0.7)	2.1 (1.5)	1.9 (1.5)	-0.2 (-0.6 – 0.2)	-0.35 (-1.15 – 0.44)	0.660
Vegetable servings (daily)	2.1 (1.2)	2.3 (1.1)	0.1 (-0.4 – 0.6)	3.5 (11.2)	2.2 (1.7)	-1.5 (-4.5 – 1.4)	-1.64 (-7.17 – 3.89)	0.859
Alcohol consumers, %	n=12, 57.14%	n=12, 66.67%		n=47, 60.26%	n=37, 56.06%			
Alcoholic drinks (weekly)	7.1 (5.4)	5.4 (4.1)	-1.1 (-3.6 – 1.4)	7.9 (9.2)	9.9 (17.2)	1.1 (-4.9 – 7.1)	2.20 (-8.85 – 13.25)	0.476
Current smokers, %	n=0	n=0		N=7, 8.97%	n=6, 9.09%			
Cigarettes (daily)	-	-	-	8.4 (4.3)	10.3 (10.1)	-3.6 (-9.2 – 2.0)	-	-

Abbreviations: PAM-13, Patient Activation Measure 13; PACIC, Patient Assessment of Chronic Illness Care.
AF Knowledge Scale, total score is correct responses out of 10 knowledge questions; PAM-13 total scores range from 0 to 100 (higher scores represent more patient activation in disease self-management) and are categorised into levels (level 1 = not believing activation important; level 2 = a lack of knowledge and confidence to take action; level 3 = beginning to take action, level 4 = taking action); PACIC subscales (patient activation, decision support, goal setting, problem solving and follow-up) and a total score (average of completed items) ranging from 0 to 5 (higher scores are reflective of better patient-health care team interactions and self-management support); exercise, fruit and vegetable servings, alcohol, and smoking are self-reported.
*Adjusted analysis consisted of a ANCOVA test, adjusting for baseline level to estimate the difference between groups at 6 months.

Table S5. Secondary outcomes of medication adherence, healthcare service utilisation patterns, health outcomes

	Control		Intervention		Odds ratio (95% CI)	p-value
	**Baseline**	**6 months**	**Baseline**	**6 months**		
**Medication adherence**	17 (81.0%)	17 (89.5%)	64 (82.1%)	54 (80.6%)	2.06 (0.42 – 10.4)	0.3733
**Healthcare service utilisation pattern [a]**						
GP or cardiologist visit	20 (95.2%)	13 (68.4%)	72 (92.3%)	53 (79.1%)	0.51 (0.16 – 1.64)	0.2601
Emergency department presentation/ hospitalisation	7 (33.3%)	6 (31.6%)	44 (56.4%)	20 (29.9%)	1.48 (0.46 – 4.82)	0.5111
Ablation/cardioversion procedure	10 (47.6%)	6 (31.6%)	29 (37.2%)	19 (28.4%)	1.01 (0.32 – 3.17)	0.9796
**Health outcomes [a]**						
Stroke/ myocardial infarct	1 (4.8%)	0 (0.0%)	2 (2.6%)	2 (3.0%)	-	-

Medication adherence assessed as binary outcome of adherent (self-reported as 0 doses of missed prescribed medications in past 7 days) or not
[a] Self-reported – questions were asked to participants to reflect on their patterns/health outcomes in the previous 6 months only
All analyses are logistic regression models to assess treatment effect – outcome variable was binary (yes/no) and co-variates were treatment group and associated baseline measures.

Table S6. Outreach completion for n=82 intervention group participants, where completed is indicative of ≥50% of questions are answered in the outreach.

	Original (Call/Survey)			Modified (survey)			
**Outreach**	**Call**	**Survey**	**Total**	**Survey**	**Call**	**Survey**	**Completed**
1	62	-	75.6% (62/82)	-	62	0	75.6% (62/82)
2	49	5	66.7% (54/81)	-	49	5	66.7% (54/81)
3	30	8	66.7% (38/57)	81.0% (17/21)	30	25	70.5% (55/78)
4	26	1	61.4% (27/44)	57.6% (19/33)	26	20	59.7% (46/77)
5	18	1	57.6% (19/33)	54.5% (24/44)	18	25	55.8% (43/77)
6	15	2	68.0% (17/25)	46.2% (24/52)	15	26	53.2% (41/77)
7	8	1	56.3% (9/16)	45.2% (28/62)	8	29	47.4% (37/78)
Overall	208	18	66.9% (226/338)	52.8% (112/212)	208	130	61.5% (338/550)
The original intervention outreaches could be completed via automated calls or a survey tool (triggered only if 3 failed call attempts). The modified intervention outreaches were delivered by the survey tool only. Outreaches 1-7 occurred at 24-48 hrs, 2 weeks, 1 month, 2 months, 3 months, 4 months, 5 months, respectively.							

Table S7. Engagement defined as the number of completed outreaches for n=82 intervention group participants, where completed is indicative of ≥50% of questions are answered in the outreach.

Number of completed outreaches (out of 7)	n (%)
0	7 (8.5%)
1	9 (11.0%)
2	8 (9.8%)
3	7 (8.5%)
4	10 (12.2%)
5	12 (14.6%)
6	11 (13.4%)
7	18 (22.0%)
Average, mean (SD)	4.12 (2.35)
Higher engagement (≥4)	51 (62.2%)

Table S8. Univariate logistic regression models predicting higher individual engagement (≥4 outreaches completed)

	Odds Ratio	95% CI Lower	95% CI Upper	p-value
**Age (ref <65 years)**				
≥65 years (older)	0.69	0.28	1.71	0.427
**Sex (ref = Male)**				
Female	0.49	0.18	1.29	0.146
**Ethnicity (ref = non-Caucasian)**				
Caucasian	0.94	0.34	2.62	0.907
**Education (ref = non-university graduate)**				
University graduate	0.91	0.3	2.78	0.867
**AF type (ref = paroxysmal)**				
Persistent/permanent/not specified	0.52	0.18	1.51	0.231
**Time since AF diagnosis (ref <5 years)**				
≥5 years (older diagnosis)	1.66	0.64	4.3	0.296
**CHA_2DS_2-VASC (ref <2 score)**				
≥2 score (higher stroke risk)	1.14	0.44	3	0.786
Abbreviations: AF, atrial fibrillation; CHA_2DS_2-VASC, Congestive heart failure, hypertension, age ≥75 (doubled), diabetes, stroke (doubled), vascular disease, age 65 to 74 and sex category (female); CI, confidence interval.				

Table S9. Interactions with the educational website for n=82 intervention group participants

	Original	Modified	Total
Participants visited ≥ once, n (%)	25	31	46
Mean number of visits	4.72	4.42	5.54
Total number of website visits	118	137	255
**Educational topics**			
General AF information	55	83	138
AF medicines	46	13	59
Weight management	34	12	46
Alcohol	50	20	70
Smoking	1	3	4
Physical activity	33	11	44
Blood pressure	9	5	14
Lifestyle information	31	27	58
Abbreviations: AF, atrial fibrillation			

Table S10. Outreach perceived usefulness for n=82 intervention group participants, proportion of participants that replied 'yes' to the question, "Did you find the information in this outreach helpful?" divided by participants that attempted the outreach (answered ≥1 question)

	Original (Call/Survey)			Modified (survey)			
**Outreach**	**Call**	**Survey**	**Total**	**Survey**	**Call**	**Survey**	**Completed**
1	57	-	91.9% (57/62)	-	57	-	91.9% (57/62)
2	45	5	87.7% (50/57)	-	45	5	87.7% (50/57)
3	27	6	86.8% (33/38)	88.2% (15/17)	27	21	87.3% (48/55)
4	23	1	88.9% (24/27)	84.2% (16/19)	23	17	87.0% (40/46)
5	17	1	90.0% (18/20)	87.5% (21/24)	17	22	88.6% (39/44)
6	14	2	94.1% (16/17)	91.7% (22/24)	14	24	92.7% (38/41)
7	6	1	70.0% (7/10)	86.2% (25/29)	6	26	82.1% (32/39)
Overall	189	16	89.1% (205/231)	87.6% (99/113)	189	115	88.4% (304/344)
The original intervention outreaches could be completed via automated calls or a survey tool (triggered only if 3 failed call attempts). The modified intervention outreaches were delivered by the survey tool only. Outreaches 1-7 occurred at 24-48 hrs, 2 weeks, 1 month, 2 months, 3 months, 4 months, 5 months, respectively.							
