# Supplementary material for: Conversational AI Phone Calls to Support Patients With Atrial Fibrillation: Randomized Controlled Trial
Source: JMIR Cardio. 2025 Aug 19;9:e64326. doi: 10.2196/64326 (PMC12364416; doi:10.2196/64326)
Supplement: Multimedia Appendix 2 [file cardio-v9-e64326-s002.pdf]

THE UNIVERSITY OF SYDNEY

Westmead Applied Research Centre

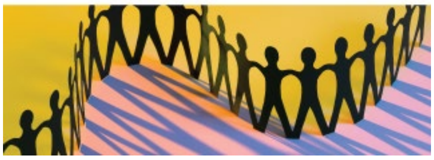

CHAT-AF

Coordinating Healthcare with Artificial Intelligence related Technology for Atrial Fibrillation Patients

STATISTICAL ANALYSIS PLAN

Prepared by: *Ritu Trivedi*

Signed by:

Investigator: *Dr Liliana Laranjo* Date: 20.10.2022

Study statistician: *Mrs Simone Marschner* Date: 20.10.2022

Chief Investigator: *Prof Clara Chow* Date: 21.10.2022

STATISTICAL ANALYSIS PLAN (SAP)

Coordinating **H**ealthcare with **A**rtificial Intelligence-supported **T**echnology in **A**trial **F**ibrillation patients: **[CHAT-AF]**

1. INTRODUCTION

This document describes the intended statistical analyses to be performed on data collected in the CHAT-AF trial. It describes, in detail, the data and variables to be summarized and analysed, including specifics of the statistical analyses to be performed. Trial registration: ACTRN12621000174886. The published protocol paper describes the CHAT-AF study in more detail.[1]

It is intended to be stand-alone from the protocol and adhere to the main points in the analysis summary specified in the protocol. However, the Statistical Analysis Plan can undergo revision outside of the protocol.

The analysis plan also outlines the proposed layout of tables and figures that will be presented.

1.1. Primary Objective

The primary objective of the study is to determine the impact of the intervention on AF-related quality of life measured by the Atrial Fibrillation Effect on QualiTy-of-life (AFEQT) questionnaire.

1.2. Secondary Objectives

1. Determine the impact of the intervention on patient-reported outcome measures: **patient activation**, measured by the Patient Activation Measure (PAM)-13 questionnaire
2. Determine the impact of the intervention on **AF knowledge** (Atrial Fibrillation Knowledge Scale) and **medication adherence**
3. Determine the impact of the intervention on patient-reported **experience measures**, namely the Patient Assessment of Chronic Illness Care (PACIC) questionnaire
4. Determine the impact of the intervention on **health outcomes** (stroke, myocardial infarct, mortality) and **healthcare service utilisation** (General Practice visits or contacts, Cardiology consultations, presentation to the ED and/or unplanned hospitalisation)
5. Determine the impact of the intervention on **lifestyle behavioural outcomes** (e.g. number of cigarettes daily, alcohol drinks per week, exercise time, BMI, servings of fruit and vegetables)
6. To assess **feasibility** with respect to potential for wider scale implementation including acceptability, perceived utility (demand), implementation and integration into IT systems (barriers and enablers) through the conduct of surveys (PACIC) and qualitative data capture from patients and healthcare providers.
7. To explore the feasibility of IVR to capture patient-reported risk assessments (e.g. overall health status, AF symptom score information) and knowledge assessments (e.g. stroke).

Secondary objectives (6) and (7) are outside the scope of the current SAP.

Outcome	Definition
**Primary**	
AFEQT total score at 6-months	The overall AFEQT score is calculated using a total of the symptoms, daily activity, and treatment concern domain scores. Scores range from 0 to 100 (higher scores associated with better health-related-QoL).
**Secondary**	
AFEQT total score at 3-month and AFEQT domain scores at 3 and 6-months	The overall AFEQT score is calculated using a total of the symptoms, daily activity, and treatment concern domain scores. Scores range from 0 to 100 (higher scores associated with better health-related-QoL). AFEQT domain scores (Symptoms, daily activities, treatment concerns, treatment satisfaction)
BMI	Calculated as weight (kg)/ height (m^2).
Number of cigarettes smoked daily	Self-reported. “How many cigarettes do you smoke per day?”
Number of alcoholic drinks consumed per week	Self-reported. “How many standard drinks of alcohol do you drink on average per week?”
Exercise minutes per week	Self-reported. “In a typical week, how much time do you spend exercising? (This includes brisk walking, swimming, yoga, skipping, cycling, sporting activities)”
Daily fruit servings	Self-reported. “How many serves of fruit do you usually eat per day?”. Definition is one serve = medium piece or two small pieces of fruit or one cup of diced pieces.
Daily vegetable servings	Self-reported. “How many serves of vegetables do you usually eat per day?”. Definition is (one serve = 1/2 cup cooked vegetables or 1 cup of salad vegetables).
Medication adherence	Self-reported. “In the last 7 days, on how many days did you miss a dose of any of your prescribed medications?” Definition: Yes = 0 = adherent; No = 1-7 = non-adherent
**Health events (in last 6 months)**	
Stroke and MI rates	Definition: documented stroke or MI. Yes = if documented in eMR OR self-reported.
GP visits	Self-reported. “In the past 6 months, how many times have you seen your GP because of heart problems or heart symptoms? Please note this includes face-to-face and telephone appointments.” Definition: # of times based on self-reported questionnaire; Yes ≥ 1 number of times; No < 1
Cardiologist visits	Self-reported. “In the past 6 months, how many times have you seen a heart specialist (cardiologist)? Please include today's appointment and all telephone appointments.”
ED visits	Self-reported. “In the past 6 months, how many times have you visited the emergency department (ED) because of possible heart symptoms or problems”.
Hospitalisations	Self-reported. “In the past 6 months, how many times have you been hospitalised because of heart problems (e.g. atrial fibrillation, stroke, heart attack or heart failure)?”
Catheter ablation	Self-reported. “In the past 6 months, have you had a catheter ablation procedure to treat your atrial fibrillation? Please note a catheter ablation is a procedure that finds where your abnormal heart rhythm comes from and treats that area of the heart.” YES = self-reported OR documented on eMR
Cardioversion	Self-reported. “In the past 6 months, have you had a cardioversion procedure to treat your atrial fibrillation? Please note a cardioversion is a procedure that uses an electrical current to reset your heart rhythm.” YES = self-reported OR documented on eMR<br><br>“Please indicate how many cardioversions you have had in the past 6 months?” Yes ≥ 1 number of times; No < 1
**AF Knowledge**	
Atrial Fibrillation Knowledge Scale	The AF Knowledge Scale questionnaire will be used to assess patients’ knowledge of AF (One-point is given for each correct response with

	potential total scores ranging from 0 to 10; the last item was removed given that it referred to the 'thrombosis center' which does not exist in Australia).
**Patient activation**	
PAM-13 scores	The Patient Activation Measure (PAM) 13 evaluates the patient's perceived knowledge, skills, and confidence in self-management activities. Question responses are structured according to a Likert scale from 1 (strongly disagree) to 4 (strongly agree) and total scores range from 0 to 100 (higher scores representing more patient activation in disease self-management).
**Patient experience**	
PACIC scores	The PACIC is a 20-item questionnaire that consists of five subscales (patient activation, decision support, goal setting, problem solving and follow-up) and an overall summary score ranging from 20 to 50.
**Abbreviations:** AF, Atrial fibrillation; AFEQT, Atrial Fibrillation Effect on QualiTy-of-life questionnaire; BMI, Body Mass Index; ED, Emergency department; eMR, Electronic medical records; GP, General practioner; MI, Myocardial infarction; PACIC, Patient Assessment of Chronic Illness Care; PAM-13, Patient activation measure 13 questionnaire.	

1.3. Trial Design

CHAT-AF is a mixed-methods experimental study with a pragmatic randomised control trial (n=385) to evaluate a digital health program ('AF-Support') in supporting patients with AF compared with usual care (in a 4:1 allocation ratio, intervention: control) on the primary outcome of AF-related QoL at six months. We will collect data at baseline, three- and six- months post-recruitment in the form of validated questionnaires, AF-related medical history, program analytics (intervention group only) and semi-structured interviews (intervention group only). The 'AF-Support' program encompasses a series of automated phone calls, text messages, emails, and an education website. The published protocol paper describes the trial design in more detail.[1]

1.4. Intervention

The 'AF-Support' program will be delivered by utilising existing software that enables the programming of a complex algorithm of automated voice calls (with conversational interactive voice response capabilities), text messages and email, along with an educational website. The published protocol paper describes the intervention in more detail.[1]

1.5. Schedule of data collection

Table: Data collection schedule

Data collected	Assessment method	Baseline	3 months	6 months
AF-related QoL	AFEQT Questionnaire[2]	X	X	X
Medication adherence	Self-reported	X		X
Lifestyle behavioural outcomes	Self-reported	X		X
AF Knowledge	AF Knowledge scale Questionnaire[3]	X		X
Patient activation measures	PAM-13 Questionnaire[4]	X		X
Patient experience measures	PACIC Questionnaire[5]	X		X
Health events	Self-reported and medical records	X		X
Healthcare services utilisation	Self-reported and medical records	X		X
Feasibility, acceptability, engagement, and utility for wider-scale implementation	Semi-structured interviews and program analytics			X*
Feasibility of IVR to capture patient responses	Program analytics	X*		
Abbreviations footnote: AF, Atrial Fibrillation; AFEQT, Atrial Fibrillation Effect on QualiTy-of-life; PAM-13, Patient Activation Measure 13; PACIC, Patient Assessment of Chronic Illness Care; ED, Emergency Department; GP, General Practitioner; IVR, Interactive Voice Response. Asterisks (*) are indicative to intervention group participants only.				

1.6. Power and sample size

A sample size of 385 can detect a difference of 7 in the total score of the AFEQT questionnaire[2,6] with 80% power ($\alpha=0.05$; standard deviation 19), accounting for a dropout rate of 10%.

2. PATIENT POPULATION

AF patients from inpatient or outpatient cardiology services at Westmead Hospital.

2.1. Inclusion criterion

Patients will be eligible to participate if they:

1. Are >18 years old
2. have a documented diagnosis of AF
3. have a mobile phone that is able to receive calls
4. are able to receive text messages or email and open weblinks embedded in them, and
5. are competent with the English language as ascertained by the study researcher.

2.2. Exclusion criterion:

Participants will be excluded from the study if they:

1. are on concurrent active intervention study focused on atrial fibrillation
2. have a concomitant illness, physical impairment or mental condition which in the opinion of the study team/primary physician could interfere with the conduct of the study including outcome assessment
3. are pregnant
4. have a medical illness with anticipated life expectancy of < 3 years and
5. are unable or unwilling to provide written consent.

3. ANALYSIS

3.1. Analysis Principles

All randomised patients will be analysed according to the intention-to-treat principle i.e. according to the group (intervention or control) they were originally assigned.

Interaction of treatment effect with age, gender, ethnicity, education, CHA2DSVASc, AF duration, anti-coagulation medication will be explored, and subgroup analyses conducted if significant ($p<0.05$).

The primary variable of AF QoL at 6 months will be explored for each of the baseline covariates listed below, exploring the interaction of these subgroups with the treatment group. A log binomial regression on 6-month QoL will be fitted, adjusting for:

- Treatment group
- Covariate measured at baseline
- Interaction term of the covariate and treatment group

If the interaction p-value is less than 0.05 then subgroup analyses will be conducted for that covariate. The baseline covariates to be examined are:

- Age (>65 years)
- Gender (male vs. female)
- Ethnicity (Caucasian vs. non-Caucasian)
- Education (high (year 11 and above)/low(Year 10 and below))
- CHA2DSVASc (≥2)
- AF duration (≥5 years)
- Anti-coagulation medication

The relative risk for each group will be calculated from the above-mentioned models, including interaction p-value, and presented in a forest plot.

All statistical analyses will be performed using R (R Core Team 2020). P-values of less than 0.05 will be considered significant.

3.2. Patient Flow Chart

Figure: shows the planned consort diagram which will be presented

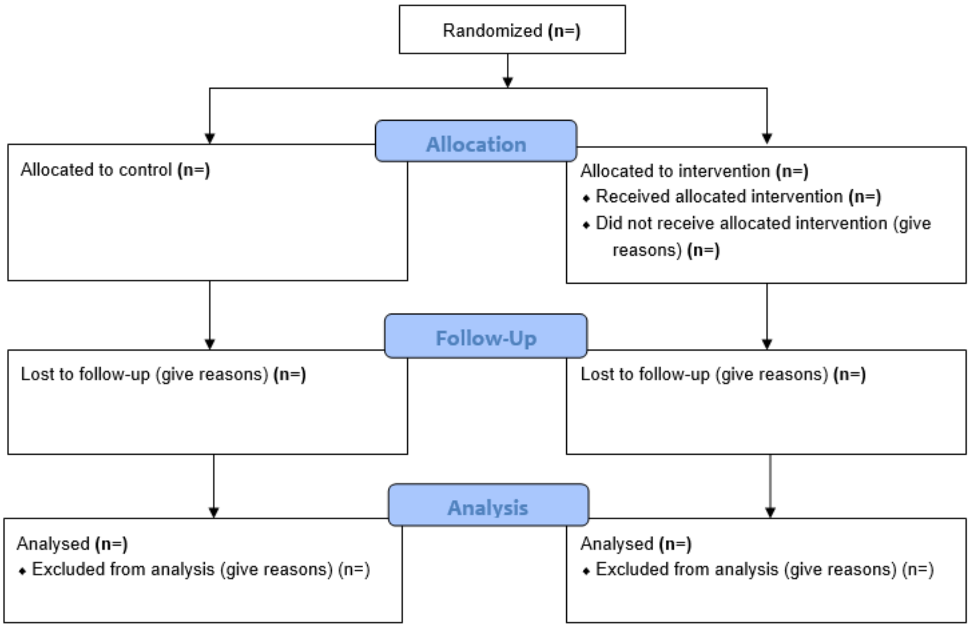

3.3. Summary of patient disposition

A summary of the number of patients randomised and reasons for not completing the study will be reported.

Table: Patient disposition

	**Control**<br>N_c (%)	**Intervention**<br>N_I (%)	**Total**<br>N (%)
**Patients randomised**	n/N_c (%)	n/N_I (%)	n/N (%)
Unable to contact	n/N_c (%)	n/N_I (%)	n/N (%)
Withdrawn	n/N_c (%)	n/N_I (%)	n/N (%)
Death	n/N_c (%)	n/N_I (%)	n/N (%)

Table: Summary of reasons for each patient not completing study

**Patient ID**	**Treatment group**	**Death/Unable to contact/withdrawn consent**	**Reason**
XX	Control	Unable to contact	
XX	Intervention	Withdrawn	
XX	Intervention	Unable to contact	

3.4. Baseline Characteristics

The cohort will be described, and treatment imbalances explored as shown in Table below.

Table: Baseline Characteristics by treatment

		**Control**<br>N_c (%)	**Intervention**<br>N_I (%)	**Total**<br>N (%)
**Sex**				
	Male	n/N_c (%)	n/N_I (%)	n/N (%)
	Female	n/N_c (%)	n/N_I (%)	n/N (%)
**Age**		Mean (SD)	Mean (SD)	Mean (SD)
	>65 years	n/N_c (%)	n/N_I (%)	n/N (%)
**Ethnicity**				
	Caucasian	n/N_c (%)	n/N_I (%)	n/N (%)
	Aboriginal/Torres Strait Islander	n/N_c (%)	n/N_I (%)	n/N (%)
	Chinese	n/N_c (%)	n/N_I (%)	n/N (%)
	Japanese	n/N_c (%)	n/N_I (%)	n/N (%)
	Malay	n/N_c (%)	n/N_I (%)	n/N (%)
	South Asian	n/N_c (%)	n/N_I (%)	n/N (%)
	Other Asian	n/N_c (%)	n/N_I (%)	n/N (%)
	Arab or Persian	n/N_c (%)	n/N_I (%)	n/N (%)
	Black African	n/N_c (%)	n/N_I (%)	n/N (%)
	Coloured African (Sub-Saharan)	n/N_c (%)	n/N_I (%)	n/N (%)
	Native American Indian	n/N_c (%)	n/N_I (%)	n/N (%)
	Other	n/N_c (%)	n/N_I (%)	n/N (%)
**Highest education**				
	None	n/N_c (%)	n/N_I (%)	n/N (%)
	Primary school	n/N_c (%)	n/N_I (%)	n/N (%)
	Year 10 School certificate	n/N_c (%)	n/N_I (%)	n/N (%)
	Year 12 Higher school certificate	n/N_c (%)	n/N_I (%)	n/N (%)
	Diploma/Technical	n/N_c (%)	n/N_I (%)	n/N (%)
	University undergraduate	n/N_c (%)	n/N_I (%)	n/N (%)
	University postgraduate	n/N_c (%)	n/N_I (%)	n/N (%)
**Household income**				
	>$15,599	n/N_c (%)	n/N_I (%)	n/N (%)
	$15,600 – $31,199	n/N_c (%)	n/N_I (%)	n/N (%)
	$31,200 – $51,999	n/N_c (%)	n/N_I (%)	n/N (%)
	$52,000 – $77,999	n/N_c (%)	n/N_I (%)	n/N (%)
	$78,000 – $104,000	n/N_c (%)	n/N_I (%)	n/N (%)

	>$104,000	n/N_c (%)	n/N_I (%)	n/N (%)
	missing	n/N_c (%)	n/N_I (%)	n/N (%)
**SBP (mmHg)**		(n=)<br>Mean (SD)	(n=)<br>Mean (SD)	(n=)<br>Mean (SD)
**DBP (mmHg)**		(n=)<br>Mean (SD)	(n=)<br>Mean (SD)	(n=)<br>Mean (SD)
**BMI**		(n=)<br>Mean (SD)	(n=)<br>Mean (SD)	(n=)<br>Mean (SD)
	BMI ≥ 25 kg/m2 (overweight)	n/N_c (%)	n/N_I (%)	n/N (%)
	BMI ≥ 30 kg/m2 (obese)	n/N_c (%)	n/N_I (%)	n/N (%)
**Smoking status**				
	Never smoked	n/N_c (%)	n/N_I (%)	n/N (%)
	Ex-smoker (not in 30 days)	n/N_c (%)	n/N_I (%)	n/N (%)
	Current smoker	n/N_c (%)	n/N_I (%)	n/N (%)
	Number of cigarettes daily (current smoker only)	(n=)<br>Mean (SD)	(n=)<br>Mean (SD)	(n=)<br>Mean (SD)
**Alcohol consumption**		n/N_c (%)	n/N_I (%)	n/N (%)
	Number of standard drinks weekly	(n=)<br>Mean (SD)	(n=)<br>Mean (SD)	(n=)<br>Mean (SD)
**Exercise (minutes/week)**		(n=)<br>Mean (SD)	(n=)<br>Mean (SD)	(n=)<br>Mean (SD)
**Diet**				
	Daily servings of fruit	(n=)<br>Mean (SD)	(n=)<br>Mean (SD)	(n=)<br>Mean (SD)
	Achieved guideline recommended ≥ 2 fruit serves	n/N_c (%)	n/N_I (%)	n/N (%)
	Daily servings of vegetables	(n=)<br>Mean (SD)	(n=)<br>Mean (SD)	(n=)<br>Mean (SD)
	Achieved guideline recommended ≥ 5 vegetable serves	n/N_c (%)	n/N_I (%)	n/N (%)
**Medication adherence**				
	Number of days medication missed in past 7 days	(n=)<br>Mean (SD)	(n=)<br>Mean (SD)	(n=)<br>Mean (SD)
	Adherent (YES=0 days missed)	n/N_c (%)	n/N_I (%)	n/N (%)
**Time since AF diagnosis**				
	<1 year	n/N_c (%)	n/N_I (%)	n/N (%)
	1-5 years	n/N_c (%)	n/N_I (%)	n/N (%)
	>5 years	n/N_c (%)	n/N_I (%)	n/N (%)
**Type of AF**				
	Paroxysmal	n/N_c (%)	n/N_I (%)	n/N (%)
	Persistent	n/N_c (%)	n/N_I (%)	n/N (%)
	Permanent	n/N_c (%)	n/N_I (%)	n/N (%)
	Unspecified	n/N_c (%)	n/N_c (%)	n/N_c (%)
**Medical History**				
	Heart Failure	n/N_c (%)	n/N_I (%)	n/N (%)
	Hypertension	n/N_c (%)	n/N_I (%)	n/N (%)
	Hyperlipidemia	n/N_c (%)	n/N_I (%)	n/N (%)
	Diabetes Mellitus Type 1	n/N_c (%)	n/N_I (%)	n/N (%)
	Diabetes Mellitus Type 2	n/N_c (%)	n/N_I (%)	n/N (%)
	Obstructive sleep apnoea	n/N_c (%)	n/N_I (%)	n/N (%)
	Depression	n/N_c (%)	n/N_I (%)	n/N (%)
	Anxiety	n/N_c (%)	n/N_I (%)	n/N (%)
	Valvular heart disease	n/N_c (%)	n/N_I (%)	n/N (%)
	Cardiomyopathy	n/N_c (%)	n/N_I (%)	n/N (%)
	Chronic renal failure	n/N_c (%)	n/N_I (%)	n/N (%)
	Peripheral vascular disease	n/N_c (%)	n/N_I (%)	n/N (%)
	Coronary artery disease	n/N_c (%)	n/N_I (%)	n/N (%)
**CHA2DS2-VASc**		(n=)<br>Mean (SD)	(n=)<br>Mean (SD)	(n=)<br>Mean (SD)
	≥2	n/N_c (%)	n/N_I (%)	n/N (%)
**Medications**				

	Antiarrhythmic	n/N_c (%)	n/N_I (%)	n/N (%)
	Anticoagulation	n/N_c (%)	n/N_I (%)	n/N (%)
	Statin	n/N_c (%)	n/N_I (%)	n/N (%)
	Angiotensin-converting-enzyme inhibitor	n/N_c (%)	n/N_I (%)	n/N (%)
	Angiotensin II receptor blocker	n/N_c (%)	n/N_I (%)	n/N (%)
	Calcium channel blocker	n/N_c (%)	n/N_I (%)	n/N (%)
	Neprilysin inhibitor	n/N_c (%)	n/N_I (%)	n/N (%)
**ED presentations***		n/N_c (%)	n/N_I (%)	n/N (%)
	Average ED presentations	(n=)<br>Mean (SD)	(n=)<br>Mean (SD)	(n=)<br>Mean (SD)
**Hospitalisations***		n/N_c (%)	n/N_I (%)	n/N (%)
	Average hospitalisations	(n=)<br>Mean (SD)	(n=)<br>Mean (SD)	(n=)<br>Mean (SD)
**Cardiologist visits***		n/N_c (%)	n/N_I (%)	n/N (%)
	Average cardiologists' visits	(n=)<br>Mean (SD)	(n=)<br>Mean (SD)	(n=)<br>Mean (SD)
**GP visits***		n/N_c (%)	n/N_I (%)	n/N (%)
	Average GP visits	(n=)<br>Mean (SD)	(n=)<br>Mean (SD)	(n=)<br>Mean (SD)
**Catheter ablation procedure***		n/N_c (%)	n/N_I (%)	n/N (%)
**Cardioversion in procedure***		n/N_c (%)	n/N_I (%)	n/N (%)
	Number of cardioversions	(n=)<br>Mean (SD)	(n=)<br>Mean (SD)	(n=)<br>Mean (SD)
**Stroke/TIA***		n/N_c (%)	n/N_I (%)	n/N (%)
**Myocardial infarction***		n/N_c (%)	n/N_I (%)	n/N (%)
**AFEQT**	**AFEQT Total score**	(n=)<br>Mean (SD)	(n=)<br>Mean (SD)	(n=)<br>Mean (SD)
	Symptom score	(n=)<br>Mean (SD)	(n=)<br>Mean (SD)	(n=)<br>Mean (SD)
	Daily activity score	(n=)<br>Mean (SD)	(n=)<br>Mean (SD)	(n=)<br>Mean (SD)
	Treatment score	(n=)<br>Mean (SD)	(n=)<br>Mean (SD)	(n=)<br>Mean (SD)
	Satisfaction score	(n=)<br>Mean (SD)	(n=)<br>Mean (SD)	(n=)<br>Mean (SD)
**AF Knowledge Scale Score (out of 10)**		(n=)<br>Mean (SD)	(n=)<br>Mean (SD)	(n=)<br>Mean (SD)
**PAM Score**	**PAM Total score**	(n=)<br>Mean (SD)	(n=)<br>Mean (SD)	(n=)<br>Mean (SD)
	level 1 (PAM≤47.0), not believing activation important	n/N_c (%)	n/N_I (%)	n/N (%)
	level 2 (PAM = 47.1 – 55.1), a lack of knowledge and confidence to take action	n/N_c (%)	n/N_I (%)	n/N (%)
	level 3 (PAM = 55.2 – 67.0),beginning to take action	n/N_c (%)	n/N_I (%)	n/N (%)
	level 4 (PAM≥67.1), taking action	n/N_c (%)	n/N_I (%)	n/N (%)
**PACIC**	**PACIC Total score**	(n=)<br>Mean (SD)	(n=)<br>Mean (SD)	(n=)<br>Mean (SD)
	Patient activation	(n=)<br>Mean (SD)	(n=)<br>Mean (SD)	(n=)<br>Mean (SD)
	Delivery system design/decision support	(n=)<br>Mean (SD)	(n=)<br>Mean (SD)	(n=)<br>Mean (SD)
	Goal setting/tailoring	(n=)<br>Mean (SD)	(n=)<br>Mean (SD)	(n=)<br>Mean (SD)
	Problem solving/Contextual	(n=)<br>Mean (SD)	(n=)<br>Mean (SD)	(n=)<br>Mean (SD)
	Follow-up/Coordination	(n=)<br>Mean (SD)	(n=)<br>Mean (SD)	(n=)<br>Mean (SD)

(*) in the last 6 months

3.5. Data completeness and distribution

3.5.1. Observation Windows

We have applied the following observation windows at baseline, three- and six- months for the below outcomes:

- Height/weight/SBP/DBP (baseline and 6-months): +/- 3 months
- 3-month questionnaires (AFEQT): -1 week and + 4 weeks
- 6-month questionnaires: -1 week and + 8 weeks

A sensitivity analysis including observations outside these windows will be performed.

3.5.2. Data availability for endpoint analysis

Table: Primary outcome data (AFEQT total score) availability for endpoint analysis by treatment group.

		**Control** N_c (%)	**Intervention** N_I (%)	**Total** N (%)
**Baseline**				
	Inside window	N=x (%)	N=x (%)	N=x (%)
	Outside window	N=x (%)	N=x (%)	N=x (%)
	Missing	N=x (%)	N=x (%)	N=x (%)
**3 months**				
	Inside window	N=x (%)	N=x (%)	N=x (%)
	Outside window	N=x (%)	N=x (%)	N=x (%)
	Missing	N=x (%)	N=x (%)	N=x (%)
**6 months**				
	Inside window	N=x (%)	N=x (%)	N=x (%)
	Outside window	N=x (%)	N=x (%)	N=x (%)
	Missing	N=x (%)	N=x (%)	N=x (%)

3.6. Primary Outcome

3.6.1. Primary outcome determination

The primary outcome is the AF-related quality of life score at 6 months measured by the Atrial Fibrillation Effect on QualiTy-of-life (AFEQT) questionnaire.

3.6.2. Primary outcomes analysis

AFEQT Total Score at baseline, 6 months and the change between baseline and 6 months will be presented by treatment group as shown below. Analysis of covariance (ANCOVA) adjusting for baseline level will be used to assess the treatment effect at 6 months.

Table: Primary analysis

	**Control** N_c	**Intervention** N_I	**Mean difference (95% CI)**	**p-value**
AFEQT Total Score at baseline	Mean (SD) 95% CI	Mean (SD) 95% CI	Difference (95% CI)	
AFEQT Total Score at 6 months	Mean (SD) 95% CI	Mean (SD) 95% CI	Difference (95% CI)	
Change in AFEQT Total Score from baseline to 6 months	Mean (SD) 95% CI	Mean (SD) 95% CI	Difference (95% CI)	
Adjusted analysis of outcomes at 6 months	Mean (SD) 95% CI	Mean (SD) 95% CI	Difference** (95% CI)	p-value
**The mean difference is estimated using the analysis of covariance with the baseline value as a covariate				

In addition, we will do an adjusted model, considering variables that are known to be associated with the QoL (e.g. age, CHA2DS2-VASc) to further explore the treatment effect on QoL at 6 months.

3.6.3. Sensitivity Analyses

In addition to the main analysis, sensitivity analyses will be performed as specified below:

- If >5% of AFEQT 6-month data is outside the observation windows prespecified above (section 3.5.1), we will do a sensitivity analysis including this data.
- If >5% of AFEQT 6-month data is missing, we will do a sensitivity analysis using the carried forward method using the 3-month AFEQT total score.
- If >5% of AFEQT 6-month data is missing, we will do a sensitivity analysis using a baseline imputation method.

3.6.4. Missing Data

We will describe the missing data at baseline and 6-month by treatment groups, and further explore whether this data is due to randomness or systematic missingness, by fitting a logistic regression model with a binary outcome of missing or not missing, with baseline covariates gender, age (>65), ethnicity (Caucasian/Non-Caucasian).

3.7. Secondary Outcomes

3.7.1. Atrial Fibrillation Effect on QualiTy-of-life (AFEQT) questionnaire: domain scores and total score at 3-months

The AFEQT questionnaire consists of 5 subscales: symptom, daily activity, treatment, satisfaction, and total score. AFEQT score of each domain at baseline, 3/6 months and the change between baseline and 3/6 months will be presented by treatment group as shown below, as well as total score at 3 months.

Table: AFEQT score of each domain score

	**Control N_c**			**Intervention N_I**			
	**Baseline**	**3/6-months**	**Difference**	**Baseline**	**3/6-months**	**Difference**	**p-value***
**Symptom**	Mean (SD)	Mean (SD)	Mean (SD)	Mean (SD)	Mean (SD)	Mean (SD)	
**Daily activity**	Mean (SD)	Mean (SD)	Mean (SD)	Mean (SD)	Mean (SD)	Mean (SD)	
**Treatment**	Mean (SD)	Mean (SD)	Mean (SD)	Mean (SD)	Mean (SD)	Mean (SD)	
**Satisfaction**	Mean (SD)	Mean (SD)	Mean (SD)	Mean (SD)	Mean (SD)	Mean (SD)	
**Total Score**	Mean (SD)	Mean (SD)	Mean (SD)	Mean (SD)	Mean (SD)	Mean (SD)	
(*) Analysis of covariance (ANCOVA) adjusting for baseline level will be used to estimate the difference between groups at 6 months.							

3.7.2. AF Knowledge

The results of the AF knowledge questionnaire recorded at baseline and 6 months are summarised in the table below.

Table: AF knowledge analysis for each question

		Baseline		6 months	
	**Correct Answer**	Control N_c	Intervention N_I	Control N_c	Intervention N_I
1.What are trigger factors for atrial fibrillation?	(B) Alcohol, coffee or spicy food	n/N_c (%)	n/N_I (%)	n/N_c (%)	n/N_I (%)
2.Why is it important to take my medication for atrial fibrillation properly?	(B) To prevent severe consequences of the arrhythmia	n/N_c (%)	n/N_I (%)	n/N_c (%)	n/N_I (%)
3.If atrial fibrillation is identified without the patient experiencing any complaints, the patient should immediately visit the hospital.	(A) True	n/N_c (%)	n/N_I (%)	n/N_c (%)	n/N_I (%)
4.What is atrial fibrillation?	(C) An electric disorder in the atria of the heart which results in the heart contracting too fast and irregularly	n/N_c (%)	n/N_I (%)	n/N_c (%)	n/N_I (%)
5.Why is oral anticoagulation medication prescribed in certain patients with atrial fibrillation?	(A) To prevent the risk of blood clots which can cause a stroke	n/N_c (%)	n/N_I (%)	n/N_c (%)	n/N_I (%)
6.Why should a person using anticoagulation medication be careful with the use of alcohol?	(A) Alcohol increases the retention of fluid in the body resulting in the blood becoming too thin	n/N_c (%)	n/N_I (%)	n/N_c (%)	n/N_I (%)
7.Atrial fibrillation is a rare condition.	(B) False	n/N_c (%)	n/N_I (%)	n/N_c (%)	n/N_I (%)
8.It is particularly risky if a person does not feel his/her atrial fibrillation.	(A) True	n/N_c (%)	n/N_I (%)	n/N_c (%)	n/N_I (%)
9.Which statement with regards to physical exercise is true for patients with atrial fibrillation?	(C) It is important to exercise normally within personal limitations	n/N_c (%)	n/N_I (%)	n/N_c (%)	n/N_I (%)
10.Which statement is true?	(C) Atrial fibrillation is harmless if the right medication is taken	n/N_c (%)	n/N_I (%)	n/N_c (%)	n/N_I (%)

AF knowledge score (questions answered correctly out of 10) at baseline, 6 months, the change between baseline and 6 months will be presented by treatment group as shown below.

Table: AF knowledge analysis

	**Control N_c**			**Intervention N_I**			
	**Baseline**	**6-months**	**Difference**	**Baseline**	**6-months**	**Difference**	**p-value***
**Total Score (out of 10)**	score (SD)	score (SD)	score (SD)	score (SD)	score (SD)	score (SD)	
(*) Analysis of covariance (ANCOVA) adjusting for baseline level will be used to estimate the difference between groups at 6 months.							

3.7.3. *Patient activation (PAM 13)*

The Patient Activation Measure® (PAM®) Survey is used to measure patient's active behaviour in the self-management of chronic illness. It is a 13-item scale with a 5-point Likert response scale (Disagree strongly, disagree, agree, agree strongly, or N/A). Raw scores are transformed (based on calibration tables) to a scaled of 0-100 with 100 being the highest activation in self-management.

Each item rated on 4-point scale ("Totally Disagree" = 1, "Disagree" = 2, "Agree" = 3, "Totally Agree" = 4, with additional "not applicable" option)

Patient Activation Measure- 13 items:

1. When all is said and done, I am the person who is responsible for taking care of my health
2. Taking an active role in my own health care is the most important thing that affects my health
3. I am confident I can help prevent or reduce problems associated with my health
4. I know what each of my prescribed medications do
5. I am confident that I can tell whether I need to go to the doctor or whether I can take care of a health problem myself
6. I am confident that I can tell a doctor concerns I have even when he or she does not ask
7. I am confident that I can follow through on medical treatments I may need to do at home
8. I understand my health problems and what causes them
9. I know what treatments are available for my health problems
10. I have been able to maintain (keep up with) lifestyle changes, like eating right or exercising
11. I know how to prevent problems with my health
12. I am confident I can figure out solutions when new problems arise with my health
13. I am confident that I can maintain lifestyle changes, like eating right and exercising, even during times of stress

PAM score (0=worst to 100=best) at baseline, 6 months, the change between baseline and 6 months will be presented by treatment group as shown below.

Table: Overall PAM score analysis

	**Control N_c**			**Intervention N_I**			
	**Baseline**	**6-months**	**Difference**	**Baseline**	**6-months**	**Difference**	**p-value***
**PAM Total Score (out of 100)**	score (SD)	score (SD)	score (SD)	score (SD)	score (SD)	score (SD)	
(*) Analysis of covariance (ANCOVA) adjusting for baseline level will be used to estimate the difference between groups at 6 months.							

Raw scores can be converted into activation levels which can also be used as cut-offs: level 1 = ≤47.0, not believing activation important; level 2 = 47.1–55.1, a lack of knowledge and confidence to take action; level 3 = 55.2–67.0, beginning to take action, level 4 = ≥67.1, taking action.

A chi-squared test on 6 months by PAM categories will be performed.

Table: Categorical PAM score analysis

	**Baseline**		**6 months**	
	Control N_c	Intervention N_I	Control N_c	Intervention N_I
PAM Level 1	n/N_c (%)	n/N_I (%)	n/N_c (%)	n/N_I (%)
PAM Level 2	n/N_c (%)	n/N_I (%)	n/N_c (%)	n/N_I (%)
PAM Level 3	n/N_c (%)	n/N_I (%)	n/N_c (%)	n/N_I (%)
PAM Level 4	n/N_c (%)	n/N_I (%)	n/N_c (%)	n/N_I (%)

3.7.4. Patient Assessment of Chronic Illness Care (PACIC) questionnaire

The PACIC has 5 subscales addressing the following domains:

- **Patient activation (items 1-3):** Actions that solicit patient input and involvement in decision-making
- **Delivery system design/decision support (items 4-6):** Actions that organize care and provide information to patients to enhance their understanding of care
- **Goal setting/tailoring (items 7-11):** Acquiring information for and setting of specific, collaborative goals
- **Problem solving/Contextual (items 12-15):** Considering potential barriers and the patient's social and cultural environment in making treatment plans
- **Follow-up/Coordination (items 16-20):** Arranging care that extends and reinforces office-based treatment, and making proactive contact with patients to assess progress and coordinate care

Each scale is scored by averaging the items completed within that scale, and the overall PACIC is scored by averaging scores across all 20 items. Each item was scored on a 5-point scale; 1 = almost never, 2 = generally not, 3 = sometimes, 4 = most of the time, 5 = almost always.

Overall PACIC score and subscale scores at baseline, 6 months, the change between baseline and 6 months will be presented by treatment group as shown below.

Table: Overall PACIC analysis

	**Control N_c**			**Intervention N_I**			
	**Baseline**	**6-months**	**Difference**	**Baseline**	**6-months**	**Difference**	**p-value***
Patient activation	Mean (SD)	Mean (SD)	Mean (SD)	Mean (SD)	Mean (SD)	Mean (SD)	
Delivery system design/decision support	Mean (SD)	Mean (SD)	Mean (SD)	Mean (SD)	Mean (SD)	Mean (SD)	
Goal setting/tailoring	Mean (SD)	Mean (SD)	Mean (SD)	Mean (SD)	Mean (SD)	Mean (SD)	
Problem solving/Contextual	Mean (SD)	Mean (SD)	Mean (SD)	Mean (SD)	Mean (SD)	Mean (SD)	
Follow-up/Coordination	Mean (SD)	Mean (SD)	Mean (SD)	Mean (SD)	Mean (SD)	Mean (SD)	
**Total PACIC**	Mean (SD)	Mean (SD)	Mean (SD)	Mean (SD)	Mean (SD)	Mean (SD)	
(*) Analysis of covariance (ANCOVA) adjusting for baseline level will be used to estimate the difference between groups at 6 months.							

3.7.5. Lifestyle behavioural outcomes

All variables below were self-reported via study-specific questions:

- Number of cigarettes smoked daily: "How many cigarettes do you smoke per day?"
- Number of alcoholic drinks consumed per week: "How many standard drinks of alcohol do you drink on average per week?"
- Exercise minutes per week: "In a typical week, how much time do you spend exercising? (This includes brisk walking, swimming, yoga, skipping, cycling, sporting activities)"
- Daily fruit servings "How many serves of fruit do you usually eat per day?". Definition is one serve = medium piece or two small pieces of fruit or one cup of diced pieces.
- Daily vegetable servings: "How many serves of vegetables do you usually eat per day?". Definition is (one serve = 1/2 cup cooked vegetables or 1 cup of salad vegetables).

Lifestyle behavioural outcomes at baseline, 6 months, the change between baseline and 6 months will be presented by treatment group as shown below.

Table: Lifestyle data analysis

	**Control** N_c			**Intervention** N_I			
	**Baseline**	**6-months**	**Difference**	**Baseline**	**6-months**	**Difference**	**P-value ***
BMI	mean (SD)	mean (SD)	mean (SD)	mean (SD)	mean (SD)	mean (SD)	
Number of cigarettes smoked daily	Median (IQR)	Median (IQR)	Median (IQR)	Median (IQR)	Median (IQR)	Median (IQR)	
Alcoholic drinks consumed weekly	Median (IQR)	Median (IQR)	Median (IQR)	Median (IQR)	Median (IQR)	Median (IQR)	
Exercise minutes per week	Median (IQR)	Median (IQR)	Median (IQR)	Median (IQR)	Median (IQR)	Median (IQR)	
Daily fruit servings	Median (IQR)	Median (IQR)	Median (IQR)	Median (IQR)	Median (IQR)	Median (IQR)	
Daily vegetable servings	Median (IQR)	Median (IQR)	Median (IQR)	Median (IQR)	Median (IQR)	Median (IQR)	
(*) Analysis of covariance (ANCOVA) adjusting for baseline level will be used to estimate the difference between groups at 6 months.							

3.7.6. *Medication adherence*

Medication adherence was self-reported, the question asked was "In the last 7 days, on how many days did you miss a dose of any of your prescribed medications?"

A logistic regression model will be fitted adjusting for baseline to assess the treatment effect on medication adherence. Definition: Yes = 0 days = adherent; No = 1-7 days = non-adherent.

Table: medical adherence analysis

	**Control** N_c	**Intervention** N_I	**Odds Ratio (95% CI)**
Adherent at baseline	n/N_c (%)	n/N_T (%)	
Adherent at 6-months	n/N_c (%)	n/N_T (%)	OR (95% CI)

3.7.7. *Health outcomes and healthcare services use.*

The OR and 95% CI will be presented for the following variables:

- Stroke and MI rates: documented stroke or MI in the past 6 months. YES = self-reported OR documented on eMR
- GP visits: "In the past 6 months, how many times have you seen your GP because of heart problems or heart symptoms? Please note this includes face-to-face and telephone appointments." Definition: # of times based on self-reported questionnaire; Yes ≥ 1 number of times; No ≤ 1
- Cardiologist visits: "In the past 6 months, how many times have you seen a heart specialist (cardiologist)? Please include today's appointment and all telephone appointments."
- ED visits: "In the past 6 months, how many times have you visited the emergency department (ED) because of possible heart symptoms or problems".
- Hospitalisations: "In the past 6 months, how many times have you been hospitalised because of heart problems (e.g. atrial fibrillation, stroke, heart attack or heart failure)?"
- Catheter ablation: "In the past 6 months, have you had a catheter ablation procedure to treat your atrial fibrillation? Please note a catheter ablation is a procedure that finds where your abnormal heart rhythm comes from and treats that area of the heart." YES = self-reported OR documented on eMR
- Cardioversion Self-reported. "In the past 6 months, have you had a cardioversion procedure to treat your atrial fibrillation? Please note a cardioversion is a procedure that uses an electrical current to reset your heart rhythm." YES = self-reported OR documented on eMR
    - "Please indicate how many cardioversions you have had in the past 6 months?"

Binary outcomes, Fisher test (if less than 5 per cell)

Table: Health outcomes and healthcare use analysis

	**Control**	**Treatment**	**Odds Ratio (95% CI)**
**Health events (Stoke/TIA/MI)**	n/N_c (%)	n/N_T (%)	OR (95% CI)
**Health service utilisation**			
GP/Cardiologist visit	n/N_c (%)	n/N_T (%)	OR (95% CI)
ED/Hospitalisation	n/N_c (%)	n/N_T (%)	OR (95% CI)
**Procedures**	n/N_c (%)	n/N_T (%)	OR (95% CI)
Ablation	n/N_c (%)	n/N_T (%)	OR (95% CI)
Cardioversion	n/N_c (%)	n/N_T (%)	OR (95% CI)

References:

1. Laranjo, L., *et al.* Coordinating Health Care With Artificial Intelligence–Supported Technology for Patients With Atrial Fibrillation: Protocol for a Randomized Controlled Trial. *JMIR research protocols* **11**, e34470 (2022).
2. Spertus, J. Development and validation of the Atrial Fibrillation Effect on QualiTy-of-Life (AFEQT) Questionnaire in patients with atrial fibrillation. *Circulation.* **4**, 15 (2011).
3. Hendriks, J.M.L. The atrial fibrillation knowledge scale: development, validation and results. *International journal of cardiology.* **168**, 1422 (2013).
4. Hibbard, J.H. Development and testing of a short form of the patient activation measure. *Health services research.* **40**, 1918 (2005).
5. Glasgow, R.E., *et al.* Development and validation of the patient assessment of chronic illness care (PACIC). *Medical care*, 436-444 (2005).
6. Mark, D.B. Effect of catheter ablation vs medical therapy on quality of life among patients with atrial fibrillation: the CABANA randomized clinical trial. *JAMA : the journal of the American Medical Association.* **321**, 1275 (2019).
