# Supplementary material for: Conversational AI Phone Calls to Support Patients With Atrial Fibrillation: Randomized Controlled Trial
Source: JMIR Cardio. 2025 Aug 19;9:e64326. doi: 10.2196/64326 (PMC12364416; doi:10.2196/64326)
Supplement: Checklist 1 [file cardio-v9-e64326-s003.pdf]

CONSORT-EHEALTH (V 1.6.1) - Submission/Publication Form

The CONSORT-EHEALTH checklist is intended for authors of randomized trials evaluating web-based and Internet-based applications/interventions, including mobile interventions, electronic games (incl multiplayer games), social media, certain telehealth applications, and other interactive and/or networked electronic applications. Some of the items (e.g. all subitems under item 5 - description of the intervention) may also be applicable for other study designs.

The goal of the CONSORT EHEALTH checklist and guideline is to be
a) a guide for reporting for authors of RCTs,
b) to form a basis for appraisal of an ehealth trial (in terms of validity)

CONSORT-EHEALTH items/subitems are MANDATORY reporting items for studies published in the Journal of Medical Internet Research and other journals / scientific societies endorsing the checklist.

Items numbered 1., 2., 3., 4a., 4b etc are original CONSORT or CONSORT-NPT (non-pharmacologic treatment) items.
Items with Roman numerals (i., ii, iii, iv etc.) are CONSORT-EHEALTH extensions/clarifications.

As the CONSORT-EHEALTH checklist is still considered in a formative stage, we would ask that you also RATE ON A SCALE OF 1-5 how important/useful you feel each item is FOR THE PURPOSE OF THE CHECKLIST and reporting guideline (optional).

Mandatory reporting items are marked with a red *.
In the textboxes, either copy & paste the relevant sections from your manuscript into this form - please include any quotes from your manuscript in QUOTATION MARKS,
or answer directly by providing additional information not in the manuscript, or elaborating on why the item was not relevant for this study.

YOUR ANSWERS WILL BE PUBLISHED AS A SUPPLEMENTARY FILE TO YOUR PUBLICATION IN JMIR AND ARE CONSIDERED PART OF YOUR PUBLICATION (IF ACCEPTED).
Please fill in these questions diligently. Information will not be copyedited, so please use proper spelling and grammar, use correct capitalization, and avoid abbreviations.

DO NOT FORGET TO SAVE AS PDF _AND_ CLICK THE SUBMIT BUTTON SO YOUR ANSWERS ARE IN OUR DATABASE !!!

Citation Suggestion (if you append the pdf as Appendix we suggest to cite this paper in the caption):
Eysenbach G, CONSORT-EHEALTH Group

CONSORT-EHEALTH: Improving and Standardizing Evaluation Reports of Web-based and Mobile Health Interventions
J Med Internet Res 2011;13(4):e126
URL: http://www.jmir.org/2011/4/e126/
doi: 10.2196/jmir.1923
PMID: 22209829

Sign in to Google to save your progress. Learn more

* Indicates required question

Your name *

First Last

Ritu Trivedi

Primary Affiliation (short), City, Country *

University of Toronto, Toronto, Canada

The University of Sydney, Sydney, Australia

Your e-mail address *

abc@gmail.com

ritu.trivedi@sydney.edu.au

Title of your manuscript *

Provide the (draft) title of your manuscript.

Conversational artificial intelligence phone calls to support patients with atrial fibrillation: a randomised controlled trial

Name of your App/Software/Intervention *

If there is a short and a long/alternate name, write the short name first and add the long name in brackets.

AF-Support

Evaluated Version (if any)

e.g. "V1", "Release 2017-03-01", "Version 2.0.27913"

Your answer

Language(s) *

What language is the intervention/app in? If multiple languages are available, separate by comma (e.g. "English, French")

English

URL of your Intervention Website or App

e.g. a direct link to the mobile app on app in appstore (itunes, Google Play), or URL of the website. If the intervention is a DVD or hardware, you can also link to an Amazon page.

Your answer

URL of an image/screenshot (optional)

Your answer

Accessibility *

Can an enduser access the intervention presently?

- ○ access is free and open
- ○ access only for special usergroups, not open
- ○ access is open to everyone, but requires payment/subscription/in-app purchases
- ◉ app/intervention no longer accessible
- ○ Other:

Primary Medical Indication/Disease/Condition *

e.g. "Stress", "Diabetes", or define the target group in brackets after the condition, e.g. "Autism (Parents of children with)", "Alzheimers (Informal Caregivers of)"

Atrial fibrillation

Primary Outcomes measured in trial *

comma-separated list of primary outcomes reported in the trial

Atrial fibrillation quality of life - measured by A

Secondary/other outcomes

Are there any other outcomes the intervention is expected to affect?

AF knowledge, patient activation, patient assessment of care quality and self-management support, self-reported lifestyle behaviours, medication adherence, health care service utilisation and health outcomes. Intervention engagement and satisfaction.

Recommended "Dose" *

What do the instructions for users say on how often the app should be used?

- ( ) Approximately Daily
- ( ) Approximately Weekly
- (•) Approximately Monthly
- ( ) Approximately Yearly
- ( ) "as needed"
- ( ) Other:

Approx. Percentage of Users (starters) still using the app as recommended after 3 months *

- ( ) unknown / not evaluated
- ( ) 0-10%
- ( ) 11-20%
- ( ) 21-30%
- ( ) 31-40%
- ( ) 41-50%
- (•) 51-60%
- ( ) 61-70%
- ( ) 71%-80%
- ( ) 81-90%
- ( ) 91-100%
- ( ) Other:

Overall, was the app/intervention effective? *

- ○ yes: all primary outcomes were significantly better in intervention group vs control
- ◉ partly: SOME primary outcomes were significantly better in intervention group vs control
- ○ no statistically significant difference between control and intervention
- ○ potentially harmful: control was significantly better than intervention in one or more outcomes
- ○ inconclusive: more research is needed
- ○ Other:

Article Preparation Status/Stage *

At which stage in your article preparation are you currently (at the time you fill in this form)

- ○ not submitted yet - in early draft status
- ◉ not submitted yet - in late draft status, just before submission
- ○ submitted to a journal but not reviewed yet
- ○ submitted to a journal and after receiving initial reviewer comments
- ○ submitted to a journal and accepted, but not published yet
- ○ published
- ○ Other:

Journal *

If you already know where you will submit this paper (or if it is already submitted), please provide the journal name (if it is not JMIR, provide the journal name under "other")

- ○ not submitted yet / unclear where I will submit this
- ◉ Journal of Medical Internet Research (JMIR)
- ○ JMIR mHealth and UHealth
- ○ JMIR Serious Games
- ○ JMIR Mental Health
- ○ JMIR Public Health
- ○ JMIR Formative Research
- ○ Other JMIR sister journal
- ○ Other:

Is this a full powered effectiveness trial or a pilot/feasibility trial? *

- ◉ Pilot/feasibility
- ○ Fully powered

Manuscript tracking number *

If this is a JMIR submission, please provide the manuscript tracking number under "other" (The ms tracking number can be found in the submission acknowledgement email, or when you login as author in JMIR. If the paper is already published in JMIR, then the ms tracking number is the four-digit number at the end of the DOI, to be found at the bottom of each published article in JMIR)

- ◉ no ms number (yet) / not (yet) submitted to / published in JMIR
- ○ Other:

TITLE AND ABSTRACT

1a) TITLE: Identification as a randomized trial in the title

1a) Does your paper address CONSORT item 1a? *

I.e does the title contain the phrase "Randomized Controlled Trial"? (if not, explain the reason under "other")

- (selected) yes
- Other:

1a-i) Identify the mode of delivery in the title

Identify the mode of delivery. Preferably use “web-based” and/or “mobile” and/or “electronic game” in the title. Avoid ambiguous terms like “online”, “virtual”, “interactive”. Use “Internet-based” only if Intervention includes non-web-based Internet components (e.g. email), use “computer-based” or “electronic” only if offline products are used. Use “virtual” only in the context of “virtual reality” (3-D worlds). Use “online” only in the context of “online support groups”. Complement or substitute product names with broader terms for the class of products (such as “mobile” or “smart phone” instead of “iphone”), especially if the application runs on different platforms.

	1	2	3	4	5	
subitem not at all important	○	○	○	○	○	essential

Does your paper address subitem 1a-i? *

Copy and paste relevant sections from manuscript title (include quotes in quotation marks "like this" to indicate direct quotes from your manuscript), or elaborate on this item by providing additional information not in the ms, or briefly explain why the item is not applicable/relevant for your study

Conversational artificial intelligence phone calls to support patients with atrial fibrillation: a randomised controlled trial

1a-ii) Non-web-based components or important co-interventions in title

Mention non-web-based components or important co-interventions in title, if any (e.g., “with telephone support”).

	1	2	3	4	5	
subitem not at all important	○	○	○	○	○	essential

Does your paper address subitem 1a-ii?

Copy and paste relevant sections from manuscript title (include quotes in quotation marks "like this" to indicate direct quotes from your manuscript), or elaborate on this item by providing additional information not in the ms, or briefly explain why the item is not applicable/relevant for your study

Your answer

1a-iii) Primary condition or target group in the title

Mention primary condition or target group in the title, if any (e.g., “for children with Type I Diabetes”) Example: A Web-based and Mobile Intervention with Telephone Support for Children with Type I Diabetes: Randomized Controlled Trial

	1	2	3	4	5	
subitem not at all important	○	○	○	○	○	essential

Does your paper address subitem 1a-iii? *

Copy and paste relevant sections from manuscript title (include quotes in quotation marks "like this" to indicate direct quotes from your manuscript), or elaborate on this item by providing additional information not in the ms, or briefly explain why the item is not applicable/relevant for your study

Patient with atrial fibrillation

1b) ABSTRACT: Structured summary of trial design, methods, results, and conclusions

NPT extension: Description of experimental treatment, comparator, care providers, centers, and blinding status.

1b-i) Key features/functionalities/components of the intervention and comparator in the METHODS section of the ABSTRACT

Mention key features/functionalities/components of the intervention and comparator in the abstract. If possible, also mention theories and principles used for designing the site. Keep in mind the needs of systematic reviewers and indexers by including important synonyms. (Note: Only report in the abstract what the main paper is reporting. If this information is missing from the main body of text, consider adding it)

	1	2	3	4	5	
subitem not at all important	○	○	○	○	○	essential

Does your paper address subitem 1b-i? *

Copy and paste relevant sections from the manuscript abstract (include quotes in quotation marks "like this" to indicate direct quotes from your manuscript), or elaborate on this item by providing additional information not in the ms, or briefly explain why the item is not applicable/relevant for your study

Intervention key features, functionalities and components are mentioned - "The six-month intervention comprised of fully automated conversational AI phone calls (with speech recognition and natural language processing) that regularly assessed patient health and symptoms, and provided self-management support and education. These phone calls were supplemented with an online survey (sent via text message or email) containing replicated call content when participants could not be reached after three call attempts. If participant responses were concerning (e.g., poor overall health, low medication confidence, high symptom burden), they would be followed up with an ad hoc phone call and directed to clinical care if required. A semi-personalised online education website was also available as part of the intervention and participants were encouraged weekly (nudges delivered via text messages or emails) to visit it."

Comparator is usual care and is mentioned - "AF patients (18 years and older) were recruited post-discharge from Westmead Hospital (Sydney, Australia) cardiology services and randomised to receive either the intervention or usual care"

1b-ii) Level of human involvement in the METHODS section of the ABSTRACT

Clarify the level of human involvement in the abstract, e.g., use phrases like “fully automated” vs. “therapist/nurse/care provider/physician-assisted” (mention number and expertise of providers involved, if any). (Note: Only report in the abstract what the main paper is reporting. If this information is missing from the main body of text, consider adding it)

	1	2	3	4	5	
subitem not at all important	○	○	○	○	○	essential

Does your paper address subitem 1b-ii?

Copy and paste relevant sections from the manuscript abstract (include quotes in quotation marks "like this" to indicate direct quotes from your manuscript), or elaborate on this item by providing additional information not in the ms, or briefly explain why the item is not applicable/relevant for your study

The intervention was fully automated with ad hoc phone calls when participant responses to questions were concerning (e.g., poor overall health, low medication confidence, high symptom burden), this is mentioned in the abstract - "The six-month intervention comprised of fully automated conversational AI phone calls (with speech recognition and natural language processing) that regularly assessed patient health and symptoms, and provided self-management support and education. These phone calls were supplemented with an online survey (sent via text message or email) containing replicated call content when participants could not be reached after three call attempts. If participant responses were concerning (e.g., poor overall health, low medication confidence, high symptom burden), they would be followed up with an ad hoc phone call and directed to clinical care if required. A semi-personalised online education website was also available as part of the intervention and participants were encouraged weekly (nudges delivered via text messages or emails) to visit it."

1b-iii) Open vs. closed, web-based (self-assessment) vs. face-to-face assessments in the METHODS section of the ABSTRACT

Mention how participants were recruited (online vs. offline), e.g., from an open access website or from a clinic or a closed online user group (closed usergroup trial), and clarify if this was a purely web-based trial, or there were face-to-face components (as part of the intervention or for assessment). Clearly say if outcomes were self-assessed through questionnaires (as common in web-based trials). Note: In traditional offline trials, an open trial (open-label trial) is a type of clinical trial in which both the researchers and participants know which treatment is being administered. To avoid confusion, use “blinded” or “unblinded” to indicated the level of blinding instead of “open”, as “open” in web-based trials usually refers to “open access” (i.e. participants can self-enrol). (Note: Only report in the abstract what the main paper is reporting. If this information is missing from the main body of text, consider adding it)

	1	2	3	4	5	
subitem not at all important	○	○	○	○	○	essential

Does your paper address subitem 1b-iii?

Copy and paste relevant sections from the manuscript abstract (include quotes in quotation marks "like this" to indicate direct quotes from your manuscript), or elaborate on this item by providing additional information not in the ms, or briefly explain why the item is not applicable/relevant for your study

Your answer

1b-iv) RESULTS section in abstract must contain use data

Report number of participants enrolled/assessed in each group, the use/uptake of the intervention (e.g., attrition/adherence metrics, use over time, number of logins etc.), in addition to primary/secondary outcomes. (Note: Only report in the abstract what the main paper is reporting. If this information is missing from the main body of text, consider adding it)

	1	2	3	4	5	
subitem not at all important	○	○	○	○	○	essential

Does your paper address subitem 1b-iv?

Copy and paste relevant sections from the manuscript abstract (include quotes in quotation marks "like this" to indicate direct quotes from your manuscript), or elaborate on this item by providing additional information not in the ms, or briefly explain why the item is not applicable/relevant for your study

These results are mentioned in the abstract - "A total of 103 patients (mean age, 63.7 years [SD 11.2]; 70% male) were randomised (82 intervention); the target sample size was 385. The difference in the AFEQT total score was non-significant (adjusted mean difference 2.08, 95% CI: -7.79–11.96, p=0.464). An exploratory pre-post comparison demonstrated significant improvement in total AFEQT score in the intervention group (baseline: 70.3, 95% CI: 63.0–77.5; 6 months: 74.9, 95% CI: 64.4–85.5; p=0.337). Intervention satisfaction (88.4%) and engagement (average of 4.12 of 7 outreaches completed) were high."

1b-v) CONCLUSIONS/DISCUSSION in abstract for negative trials

Conclusions/Discussions in abstract for negative trials: Discuss the primary outcome - if the trial is negative (primary outcome not changed), and the intervention was not used, discuss whether negative results are attributable to lack of uptake and discuss reasons. (Note: Only report in the abstract what the main paper is reporting. If this information is missing from the main body of text, consider adding it)

	1	2	3	4	5	
subitem not at all important	○	○	○	○	○	essential

Does your paper address subitem 1b-v?

Copy and paste relevant sections from the manuscript abstract (include quotes in quotation marks "like this" to indicate direct quotes from your manuscript), or elaborate on this item by providing additional information not in the ms, or briefly explain why the item is not applicable/relevant for your study

The conclusions are explained in the abstract - "This proof-of-concept study demonstrates the feasibility of conversational AI in supporting patients with chronic conditions post-discharge. Intervention participants had improvement in their AF- quality of life, though the forced shortening of the evaluation was unable to demonstrate a significant difference between groups. "

INTRODUCTION

2a) In INTRODUCTION: Scientific background and explanation of rationale

2a-i) Problem and the type of system/solution

Describe the problem and the type of system/solution that is object of the study: intended as stand-alone intervention vs. incorporated in broader health care program? Intended for a particular patient population? Goals of the intervention, e.g., being more cost-effective to other interventions, replace or complement other solutions? (Note: Details about the intervention are provided in “Methods” under 5)

	1	2	3	4	5	
subitem not at all important	○	○	○	○	○	essential

Does your paper address subitem 2a-i? *

Copy and paste relevant sections from the manuscript (include quotes in quotation marks "like this" to indicate direct quotes from your manuscript), or elaborate on this item by providing additional information not in the ms, or briefly explain why the item is not applicable/relevant for your study

Background and rationales are clearly outlined - "The increase in atrial fibrillation (AF) prevalence is a global public health concern. AF presents the health system with various challenges: its rapidly increasing patient population[1], the multifaceted care required to manage patients and prevent outcomes of stroke and mortality[2], and the significant costs associated with hospitalizations[3]. Guidelines suggest a digitally-enabled integrated approach to AF management, involving a multidisciplinary team to provide patient-centred care and support patient self-management (e.g., lifestyle behaviour change, medication adherence)[2].

Existing trials of digital interventions to support AF self-management have found mixed results, with some evidence of improvements in quality of life (QoL), knowledge, medication adherence, and clinical outcomes (composite outcome comprising stroke or thromboembolism, all-cause death, and rehospitalization) compared to usual care[4-6]. The majority of digital interventions for patients with AF have primarily been delivered through mobile apps and many have reported low user engagement[7-9].

Conversational technologies now offer interactive ways of providing education and self-management support to patients[10]. These technologies can simulate human conversations through text or speech in an accessible and personalized manner, with users reporting high satisfaction. Recent voice-based conversational technologies leverage artificial intelligence (AI) (including speech recognition and natural language processing) to facilitate more engaging and human-like dialogues[11, 12]. There is limited literature available on the efficacy of conversation-AI interventions, and no studies have been conducted in an AF population[11-14]."

2a-ii) Scientific background, rationale: What is known about the (type of) system

Scientific background, rationale: What is known about the (type of) system that is the object of the study (be sure to discuss the use of similar systems for other conditions/diagnoses, if appropiate), motivation for the study, i.e. what are the reasons for and what is the context for this specific study, from which stakeholder viewpoint is the study performed, potential impact of findings [2]. Briefly justify the choice of the comparator.

	1	2	3	4	5	
subitem not at all important	○	○	○	○	○	essential

Does your paper address subitem 2a-ii? *

Copy and paste relevant sections from the manuscript (include quotes in quotation marks "like this" to indicate direct quotes from your manuscript), or elaborate on this item by providing additional information not in the ms, or briefly explain why the item is not applicable/relevant for your study

Existing literature on digital interventions for patients with AF and on conversational technologies is discussed - "Existing trials of digital interventions to support AF self-management have found mixed results, with some evidence of improvements in quality of life (QoL), knowledge, medication adherence, and clinical outcomes (composite outcome comprising stroke or thromboembolism, all-cause death, and rehospitalization) compared to usual care[4-6]. The majority of digital interventions for patients with AF have primarily been delivered through mobile apps and many have reported low user engagement[7-9].

Conversational technologies now offer interactive ways of providing education and self-management support to patients[10]. These technologies can simulate human conversations through text or speech in an accessible and personalized manner, with users reporting high satisfaction. Recent voice-based conversational technologies leverage artificial intelligence (AI) (including speech recognition and natural language processing) to facilitate more engaging and human-like dialogues[11, 12]. There is limited literature available on the efficacy of conversation-AI interventions, and no studies have been conducted in an AF population[11-14]."

2b) In INTRODUCTION: Specific objectives or hypotheses

Does your paper address CONSORT subitem 2b? *

Copy and paste relevant sections from the manuscript (include quotes in quotation marks "like this" to indicate direct quotes from your manuscript), or elaborate on this item by providing additional information not in the ms, or briefly explain why the item is not applicable/relevant for your study

The specific objectives of the study are clearly stated - "The aim of this proof-of-concept randomised controlled trial (RCT) was to evaluate an intervention comprising conversational AI automated phone calls, text messages and emails, and an educational website to better support patients with self-managing their AF[15]. The Coordinating Health care with Artificial intelligence–supported Technology for patients with Atrial Fibrillation (CHAT-AF) trial assessed the impact of this conversational AI intervention on AF-QoL, as well as evaluated its feasibility (engagement and satisfaction)."

METHODS

3a) Description of trial design (such as parallel, factorial) including allocation ratio

Does your paper address CONSORT subitem 3a? *

Copy and paste relevant sections from the manuscript (include quotes in quotation marks "like this" to indicate direct quotes from your manuscript), or elaborate on this item by providing additional information not in the ms, or briefly explain why the item is not applicable/relevant for your study

Study design is clearly stated - "...a single-blinded parallel RCT, with a 4:1 allocation chosen to optimize process evaluation (engagement and satisfaction with intervention)"

3b) Important changes to methods after trial commencement (such as eligibility criteria), with reasons

Does your paper address CONSORT subitem 3b? *

Copy and paste relevant sections from the manuscript (include quotes in quotation marks "like this" to indicate direct quotes from your manuscript), or elaborate on this item by providing additional information not in the ms, or briefly explain why the item is not applicable/relevant for your study

Reasons for premature stopping of trial is explained in detail in the methods section - "At the end of July 2021, we stopped recruitment as the delivery of the intervention was interrupted due to unforeseeable circumstances involving acquisition of the technology partner by another organisation. We had already recruited 82 intervention participants, and of these 20 had received all outreaches, with the remaining 62 participants being at differing stages of the intervention timeline. All participants had received at least 2 of 7 outreaches via the automated calls before premature study completion. We therefore modified the intervention, and the call content for the remaining outreaches was delivered only via survey format. This survey (delivered via REDCap) contained replicated content and questions as asked in the phone calls but required participants to click their responses. The hope had been to find another technology partner that could deliver the intervention according to our specifications, but we were unable to do this and limited by the remaining budget, we stopped the study to report findings. These changes were planned, reviewed, and approved by the trial steering committee. This trial is reported according to the CONSERVE (CONSORT and SPIRIT Extension for RCTs Revised in Extenuating Circumstances) statement[18]."

3b-i) Bug fixes, Downtimes, Content Changes

Bug fixes, Downtimes, Content Changes: ehealth systems are often dynamic systems. A description of changes to methods therefore also includes important changes made on the intervention or comparator during the trial (e.g., major bug fixes or changes in the functionality or content) (5-iii) and other “unexpected events” that may have influenced study design such as staff changes, system failures/downtimes, etc. [2].

	1	2	3	4	5	
subitem not at all important	○	○	○	○	○	essential

Does your paper address subitem 3b-i?

Copy and paste relevant sections from the manuscript (include quotes in quotation marks "like this" to indicate direct quotes from your manuscript), or elaborate on this item by providing additional information not in the ms, or briefly explain why the item is not applicable/relevant for your study

Your answer

4a) Eligibility criteria for participants

Does your paper address CONSORT subitem 4a? *

Copy and paste relevant sections from the manuscript (include quotes in quotation marks "like this" to indicate direct quotes from your manuscript), or elaborate on this item by providing additional information not in the ms, or briefly explain why the item is not applicable/relevant for your study

Eligibility and exclusion criteria are stated - "English-speaking adult patients with documented AF, who had a mobile phone were eligible. Pregnant women and those participating in another clinical trial focused on providing AF education were excluded."

4a-i) Computer / Internet literacy

Computer / Internet literacy is often an implicit “de facto” eligibility criterion - this should be explicitly clarified.

	1	2	3	4	5	
subitem not at all important	○	○	○	○	○	essential

Does your paper address subitem 4a-i?

Copy and paste relevant sections from the manuscript (include quotes in quotation marks "like this" to indicate direct quotes from your manuscript), or elaborate on this item by providing additional information not in the ms, or briefly explain why the item is not applicable/relevant for your study

Our eligibility criteria included ownership of a mobile phone to be able to receive phone calls and open website links (if receiving education website link via text message).

4a-ii) Open vs. closed, web-based vs. face-to-face assessments:

Open vs. closed, web-based vs. face-to-face assessments: Mention how participants were recruited (online vs. offline), e.g., from an open access website or from a clinic, and clarify if this was a purely web-based trial, or there were face-to-face components (as part of the intervention or for assessment), i.e., to what degree got the study team to know the participant. In online-only trials, clarify if participants were quasi-anonymous and whether having multiple identities was possible or whether technical or logistical measures (e.g., cookies, email confirmation, phone calls) were used to detect/prevent these.

	1	2	3	4	5	
subitem not at all important	○	○	○	○	○	essential

Does your paper address subitem 4a-ii? *

Copy and paste relevant sections from the manuscript (include quotes in quotation marks "like this" to indicate direct quotes from your manuscript), or elaborate on this item by providing additional information not in the ms, or briefly explain why the item is not applicable/relevant for your study

Information on recruitment is stated in the methods section - "Participants were recruited from inpatient and outpatient cardiology services at Westmead Hospital (Sydney, Australia). All participants provided informed consent (written or phone consent) and completed study assessments via electronic surveys at baseline (in hospital or sent link via text messages/email), 3- and 6- months (via link sent by text message or email). Baseline demographics and medical history were collected by participant self-report or by electronic medical records review (Multimedia Appendix 1). Questionnaires to assess primary and secondary outcomes were also completed electronically either in person (baseline - during initial hospital visit) or sent via text message/email (3- and 6-month follow-up)."

4a-iii) Information giving during recruitment

Information given during recruitment. Specify how participants were briefed for recruitment and in the informed consent procedures (e.g., publish the informed consent documentation as appendix, see also item X26), as this information may have an effect on user self-selection, user expectation and may also bias results.

	1	2	3	4	5	
subitem not at all important	○	○	○	○	○	essential

Does your paper address subitem 4a-iii?

Copy and paste relevant sections from the manuscript (include quotes in quotation marks "like this" to indicate direct quotes from your manuscript), or elaborate on this item by providing additional information not in the ms, or briefly explain why the item is not applicable/relevant for your study

Participants were given a signed copy of the participant information sheet and consent form to keep which contained details about the trial, along with a one-page (double sided) lay study information sheet describing details about the intervention. No formal training was required.

4b) Settings and locations where the data were collected

Does your paper address CONSORT subitem 4b? *

Copy and paste relevant sections from the manuscript (include quotes in quotation marks "like this" to indicate direct quotes from your manuscript), or elaborate on this item by providing additional information not in the ms, or briefly explain why the item is not applicable/relevant for your study

Information on setting is stated in the methods section - "Participants were recruited from inpatient and outpatient cardiology services at Westmead Hospital (Sydney, Australia). All participants provided informed consent (written or phone consent) and completed study assessments via electronic surveys at baseline (in hospital or sent link via text messages/email), 3- and 6- months (via link sent by text message or email). Baseline demographics and medical history were collected by participant self-report or by electronic medical records review (Multimedia Appendix 1). Questionnaires to assess primary and secondary outcomes were also completed electronically either in person (baseline - during initial hospital visit) or sent via text message/email (3- and 6-month follow-up)."

4b-i) Report if outcomes were (self-)assessed through online questionnaires

Clearly report if outcomes were (self-)assessed through online questionnaires (as common in web-based trials) or otherwise.

	1	2	3	4	5	
subitem not at all important	○	○	○	○	○	essential

Does your paper address subitem 4b-i? *

Copy and paste relevant sections from the manuscript (include quotes in quotation marks "like this" to indicate direct quotes from your manuscript), or elaborate on this item by providing additional information not in the ms, or briefly explain why the item is not applicable/relevant for your study

All surveys and questionnaires were completed electronically (REDCap) at baseline (on an iPad during hospital visit or at home via a link sent by text message/email), 3- and 6-months (at home via a link sent by text message/email).

4b-ii) Report how institutional affiliations are displayed

Report how institutional affiliations are displayed to potential participants [on ehealth media], as affiliations with prestigious hospitals or universities may affect volunteer rates, use, and reactions with regards to an intervention.(Not a required item – describe only if this may bias results)

	1	2	3	4	5	
subitem not at all important	○	○	○	○	○	essential

Does your paper address subitem 4b-ii?

Copy and paste relevant sections from the manuscript (include quotes in quotation marks "like this" to indicate direct quotes from your manuscript), or elaborate on this item by providing additional information not in the ms, or briefly explain why the item is not applicable/relevant for your study

The CHAT-AF study, Westmead Applied Research Centre, University of Sydney and the Western Sydney Local Health District logos were displayed on participant facing documents (information and consent form, lay study information sheets, surveys, and the education website).

5) The interventions for each group with sufficient details to allow replication, including how and when they were actually administered

5-i) Mention names, credential, affiliations of the developers, sponsors, and owners

Mention names, credential, affiliations of the developers, sponsors, and owners [6] (if authors/evaluators are owners or developer of the software, this needs to be declared in a “Conflict of interest” section or mentioned elsewhere in the manuscript).

	1	2	3	4	5	
subitem not at all important	○	○	○	○	○	essential

Does your paper address subitem 5-i?

Copy and paste relevant sections from the manuscript (include quotes in quotation marks "like this" to indicate direct quotes from your manuscript), or elaborate on this item by providing additional information not in the ms, or briefly explain why the item is not applicable/relevant for your study

This is mentioned in the methods section - "The technology in the intervention was provided by HMS."

5-ii) Describe the history/development process

Describe the history/development process of the application and previous formative evaluations (e.g., focus groups, usability testing), as these will have an impact on adoption/use rates and help with interpreting results.

	1	2	3	4	5	
subitem not at all important	○	○	○	○	○	essential

Does your paper address subitem 5-ii?

Copy and paste relevant sections from the manuscript (include quotes in quotation marks "like this" to indicate direct quotes from your manuscript), or elaborate on this item by providing additional information not in the ms, or briefly explain why the item is not applicable/relevant for your study

This information is provided in detail in the published protocol paper (JMIR Research Protocols) - https://researchprotocols.org/2022/4/e34470/

5-iii) Revisions and updating

Revisions and updating. Clearly mention the date and/or version number of the application/intervention (and comparator, if applicable) evaluated, or describe whether the intervention underwent major changes during the evaluation process, or whether the development and/or content was “frozen” during the trial. Describe dynamic components such as news feeds or changing content which may have an impact on the replicability of the intervention (for unexpected events see item 3b).

	1	2	3	4	5	
subitem not at all important	○	○	○	○	○	essential

Does your paper address subitem 5-iii?

Copy and paste relevant sections from the manuscript (include quotes in quotation marks "like this" to indicate direct quotes from your manuscript), or elaborate on this item by providing additional information not in the ms, or briefly explain why the item is not applicable/relevant for your study

Your answer

5-iv) Quality assurance methods

Provide information on quality assurance methods to ensure accuracy and quality of information provided [1], if applicable.

	1	2	3	4	5	
subitem not at all important	○	○	○	○	○	essential

Does your paper address subitem 5-iv?

Copy and paste relevant sections from the manuscript (include quotes in quotation marks "like this" to indicate direct quotes from your manuscript), or elaborate on this item by providing additional information not in the ms, or briefly explain why the item is not applicable/relevant for your study

The intervention was designed with input from clinicians (cardiologists and primary care physicians), consumers, and researchers with experience in self-management digital interventions for cardiovascular patient populations, this information is provided in detail in the published protocol paper (JMIR Research Protocols) - https://researchprotocols.org/2022/4/e34470/

5-v) Ensure replicability by publishing the source code, and/or providing screenshots/screen-capture video, and/or providing flowcharts of the algorithms used

Ensure replicability by publishing the source code, and/or providing screenshots/screen-capture video, and/or providing flowcharts of the algorithms used. Replicability (i.e., other researchers should in principle be able to replicate the study) is a hallmark of scientific reporting.

	1	2	3	4	5	
subitem not at all important	○	○	○	○	○	essential

Does your paper address subitem 5-v?

Copy and paste relevant sections from the manuscript (include quotes in quotation marks "like this" to indicate direct quotes from your manuscript), or elaborate on this item by providing additional information not in the ms, or briefly explain why the item is not applicable/relevant for your study

Screenshots and more details regarding the intervention are provided in the published protocol paper (JMIR Research Protocols) - https://researchprotocols.org/2022/4/e34470/ and in the Multimedia Appendix 1 of the current paper.

5-vi) Digital preservation

Digital preservation: Provide the URL of the application, but as the intervention is likely to change or disappear over the course of the years; also make sure the intervention is archived (Internet Archive, webcitation.org, and/or publishing the source code or screenshots/videos alongside the article). As pages behind login screens cannot be archived, consider creating demo pages which are accessible without login.

	1	2	3	4	5	
subitem not at all important	○	○	○	○	○	essential

Does your paper address subitem 5-vi?

Copy and paste relevant sections from the manuscript (include quotes in quotation marks "like this" to indicate direct quotes from your manuscript), or elaborate on this item by providing additional information not in the ms, or briefly explain why the item is not applicable/relevant for your study

Your answer

5-vii) Access

Access: Describe how participants accessed the application, in what setting/context, if they had to pay (or were paid) or not, whether they had to be a member of specific group. If known, describe how participants obtained “access to the platform and Internet” [1]. To ensure access for editors/reviewers/readers, consider to provide a “backdoor” login account or demo mode for reviewers/readers to explore the application (also important for archiving purposes, see vi).

	1	2	3	4	5	
subitem not at all important	○	○	○	○	○	essential

Does your paper address subitem 5-vii? *

Copy and paste relevant sections from the manuscript (include quotes in quotation marks "like this" to indicate direct quotes from your manuscript), or elaborate on this item by providing additional information not in the ms, or briefly explain why the item is not applicable/relevant for your study

The intervention was free, and participants were uploaded onto the HMS technology partner platform post recruitment and started receiving phone calls and links to the education website (via text or email) according to the intervention schedule (described in detail in the published protocol paper (JMIR Research Protocols) - https://researchprotocols.org/2022/4/e34470/ and in the Multimedia Appendix 1 of the current paper).

5-viii) Mode of delivery, features/functionalities/components of the intervention and comparator, and the theoretical framework

Describe mode of delivery, features/functionalities/components of the intervention and comparator, and the theoretical framework [6] used to design them (instructional strategy [1], behaviour change techniques, persuasive features, etc., see e.g., [7, 8] for terminology). This includes an in-depth description of the content (including where it is coming from and who developed it) [1],” whether [and how] it is tailored to individual circumstances and allows users to track their progress and receive feedback” [6]. This also includes a description of communication delivery channels and – if computer-mediated communication is a component – whether communication was synchronous or asynchronous [6]. It also includes information on presentation strategies [1], including page design principles, average amount of text on pages, presence of hyperlinks to other resources, etc. [1].

	1	2	3	4	5	
subitem not at all important	○	○	○	○	○	essential

Does your paper address subitem 5-viii? *

Copy and paste relevant sections from the manuscript (include quotes in quotation marks "like this" to indicate direct quotes from your manuscript), or elaborate on this item by providing additional information not in the ms, or briefly explain why the item is not applicable/relevant for your study

Details regarding the mode of delivery, features, functionalities and components of the intervention was provided in the methods section and Multimedia Appendix 1 of the current paper, as well as more detailed information is outlined in the published protocol paper (JMIR Research Protocols) - https://researchprotocols.org/2022/4/e34470/

5-ix) Describe use parameters

Describe use parameters (e.g., intended “doses” and optimal timing for use). Clarify what instructions or recommendations were given to the user, e.g., regarding timing, frequency, heaviness of use, if any, or was the intervention used ad libitum.

	1	2	3	4	5	
subitem not at all important	○	○	○	○	○	essential

Does your paper address subitem 5-ix?

Copy and paste relevant sections from the manuscript (include quotes in quotation marks "like this" to indicate direct quotes from your manuscript), or elaborate on this item by providing additional information not in the ms, or briefly explain why the item is not applicable/relevant for your study

The intervention schedule is outlined in the methods section and Multimedia Appendix 1 of the current paper, as well as more detailed information is outlined in the published protocol paper (JMIR Research Protocols) - https://researchprotocols.org/2022/4/e34470/

5-x) Clarify the level of human involvement

Clarify the level of human involvement (care providers or health professionals, also technical assistance) in the e-intervention or as co-intervention (detail number and expertise of professionals involved, if any, as well as “type of assistance offered, the timing and frequency of the support, how it is initiated, and the medium by which the assistance is delivered”. It may be necessary to distinguish between the level of human involvement required for the trial, and the level of human involvement required for a routine application outside of a RCT setting (discuss under item 21 – generalizability).

	1	2	3	4	5	
subitem not at all important	○	○	○	○	○	essential

Does your paper address subitem 5-x?

Copy and paste relevant sections from the manuscript (include quotes in quotation marks "like this" to indicate direct quotes from your manuscript), or elaborate on this item by providing additional information not in the ms, or briefly explain why the item is not applicable/relevant for your study

Your answer

5-xi) Report any prompts/reminders used

Report any prompts/reminders used: Clarify if there were prompts (letters, emails, phone calls, SMS) to use the application, what triggered them, frequency etc. It may be necessary to distinguish between the level of prompts/reminders required for the trial, and the level of prompts/reminders for a routine application outside of a RCT setting (discuss under item 21 – generalizability).

	1	2	3	4	5	
subitem not at all important	○	○	○	○	○	essential

Does your paper address subitem 5-xi? *

Copy and paste relevant sections from the manuscript (include quotes in quotation marks "like this" to indicate direct quotes from your manuscript), or elaborate on this item by providing additional information not in the ms, or briefly explain why the item is not applicable/relevant for your study

Three phone calls attempts were made per outreach, before a text message or email was sent with a link replicating the call content (two text/email reminders were made). Weekly nudges to visit the education website were sent via text/email.

5-xii) Describe any co-interventions (incl. training/support)

Describe any co-interventions (incl. training/support): Clearly state any interventions that are provided in addition to the targeted eHealth intervention, as ehealth intervention may not be designed as stand-alone intervention. This includes training sessions and support [1]. It may be necessary to distinguish between the level of training required for the trial, and the level of training for a routine application outside of a RCT setting (discuss under item 21 – generalizability.

	1	2	3	4	5	
subitem not at all important	○	○	○	○	○	essential

Does your paper address subitem 5-xii? *

Copy and paste relevant sections from the manuscript (include quotes in quotation marks "like this" to indicate direct quotes from your manuscript), or elaborate on this item by providing additional information not in the ms, or briefly explain why the item is not applicable/relevant for your study

No formal training for intervention use was required. Participants were given a signed copy of the participant information sheet and consent form to keep which contained details about the trial, along with a one-page (double sided) lay study information sheet describing details about the intervention.

6a) Completely defined pre-specified primary and secondary outcome measures, including how and when they were assessed

Does your paper address CONSORT subitem 6a? *

Copy and paste relevant sections from the manuscript (include quotes in quotation marks "like this" to indicate direct quotes from your manuscript), or elaborate on this item by providing additional information not in the ms, or briefly explain why the item is not applicable/relevant for your study

Primary and secondary outcome measure are clearly stated in the manuscript, along with additional details provided in Multimedia Appendix 1, Table 1.

"The primary outcome was change in AF-QoL assessed as Atrial Fibrillation Effect on QualiTy-of-life (AFEQT)[19] total score at 6 months. AFEQT total score (0-100) is an average of subscales (symptom, daily activity, treatment), with higher scores indicating better QoL. Secondary outcomes included AFEQT subscales, AF knowledge[20], patient activation[21], patient assessment of care quality and self-management support[22], self-reported lifestyle behaviours, medication adherence (days missed doses in prescription medications over the past week), health care service utilisation (GP or cardiologist visits, emergency department presentation or hospitalization, ablation or cardioversion procedure) and health outcomes (stroke or myocardial infarct). All outcomes were assessed at baseline and 6 months; AF-QoL (AFEQT) was also assessed at 3 months. A detailed list of outcomes, methods, and time of data collection are provided online (Multimedia Appendix 1).
Process evaluation outcomes for the intervention group included: outreach completion and satisfaction, and individual engagement (Multimedia Appendix 1). Outreach completion was calculated as the number of outreaches with at least half of the questions answered divided by the number of participants that received the outreach. Outreach satisfaction was defined as the number of individuals that answered, ‘Yes’ to the question ‘Did you find the information in this call/survey helpful?’ and was divided by the number of participants that attempted the outreach (answered at least one question). Individual engagement was calculated as the number of outreaches completed by the participant and categorized, where 4 or more completed outreaches (out of 7) were considered high engagement. Metrics for engagement with the educational website were also explored."

6a-i) Online questionnaires: describe if they were validated for online use and apply CHERRIES items to describe how the questionnaires were designed/deployed

If outcomes were obtained through online questionnaires, describe if they were validated for online use and apply CHERRIES items to describe how the questionnaires were designed/deployed [9].

	1	2	3	4	5	
subitem not at all important	◯	◯	◯	◯	◯	essential

Does your paper address subitem 6a-i?

Copy and paste relevant sections from manuscript text

Your answer

6a-ii) Describe whether and how “use” (including intensity of use/dosage) was defined/measured/monitored

Describe whether and how “use” (including intensity of use/dosage) was defined/measured/monitored (logins, logfile analysis, etc.). Use/adoption metrics are important process outcomes that should be reported in any ehealth trial.

	1	2	3	4	5	
subitem not at all important	◯	◯	◯	◯	◯	essential

Does your paper address subitem 6a-ii?

Copy and paste relevant sections from manuscript text

This information is provided in the methods section - "Process evaluation outcomes for the intervention group included: outreach completion and satisfaction, and individual engagement (Multimedia Appendix 1). Outreach completion was calculated as the number of outreaches with at least half of the questions answered divided by the number of participants that received the outreach. Outreach satisfaction was defined as the number of individuals that answered, ‘Yes’ to the question ‘Did you find the information in this call/survey helpful?’ and was divided by the number of participants that attempted the outreach (answered at least one question). Individual engagement was calculated as the number of outreaches completed by the participant and categorized, where 4 or more completed outreaches (out of 7) were considered high engagement. Metrics for engagement with the educational website were also explored."

6a-iii) Describe whether, how, and when qualitative feedback from participants was obtained

Describe whether, how, and when qualitative feedback from participants was obtained (e.g., through emails, feedback forms, interviews, focus groups).

	1	2	3	4	5	
subitem not at all important	○	○	○	○	○	essential

Does your paper address subitem 6a-iii?

Copy and paste relevant sections from manuscript text

Qualatative feedback was obtained during semi-structured interviews with intervention participants. The results from this analysis will be published in a separate results paper.

6b) Any changes to trial outcomes after the trial commenced, with reasons

Does your paper address CONSORT subitem 6b? *

Copy and paste relevant sections from the manuscript (include quotes in quotation marks "like this" to indicate direct quotes from your manuscript), or elaborate on this item by providing additional information not in the ms, or briefly explain why the item is not applicable/relevant for your study

No changes in trial outcomes, only intervention delivery, details are provided in the methods section - "At the end of July 2021, we stopped recruitment as the delivery of the intervention was interrupted due to unforeseeable circumstances involving acquisition of the technology partner by another organisation. We had already recruited 82 intervention participants, and of these 20 had received all outreaches, with the remaining 62 participants being at differing stages of the intervention timeline. All participants had received at least 2 of 7 outreaches via the automated calls before premature study completion. We therefore modified the intervention, and the call content for the remaining outreaches was delivered only via survey format. This survey (delivered via REDCap) contained replicated content and questions as asked in the phone calls but required participants to click their responses. The hope had been to find another technology partner that could deliver the intervention according to our specifications, but we were unable to do this and limited by the remaining budget, we stopped the study to report findings. These changes were planned, reviewed, and approved by the trial steering committee. This trial is reported according to the CONSERVE (CONSORT and SPIRIT Extension for RCTs Revised in Extenuating Circumstances) statement[18]."

7a) How sample size was determined

NPT: When applicable, details of whether and how the clustering by care provides or centers was addressed

7a-i) Describe whether and how expected attrition was taken into account when calculating the sample size

Describe whether and how expected attrition was taken into account when calculating the sample size.

	1	2	3	4	5	
subitem not at all important	○	○	○	○	○	essential

Does your paper address subitem 7a-i?

Copy and paste relevant sections from manuscript title (include quotes in quotation marks "like this" to indicate direct quotes from your manuscript), or elaborate on this item by providing additional information not in the ms, or briefly explain why the item is not applicable/relevant for your study

Sample size calculation is outlined in the methods section - "A sample size of 385 was required to detect a between-group difference of 7 in the total score of the AFEQT questionnaire with 80% power (α=.05; SD=19), accounting for a dropout rate of 10%[19, 23]. The current study was limited in detecting differences in primary outcome as we were only able to recruit 27% of the intended sample size (103 participants)."

7b) When applicable, explanation of any interim analyses and stopping guidelines

Does your paper address CONSORT subitem 7b? *

Copy and paste relevant sections from the manuscript (include quotes in quotation marks "like this" to indicate direct quotes from your manuscript), or elaborate on this item by providing additional information not in the ms, or briefly explain why the item is not applicable/relevant for your study

We did not conduct any intermin analyses or have any stopping guidelines, however, the trial was prematurely stopped due to acquisition of the technology partner by another organisation. Details are provided in the manuscript - "At the end of July 2021, we stopped recruitment as the delivery of the intervention was interrupted due to unforeseeable circumstances involving acquisition of the technology partner by another organisation. We had already recruited 82 intervention participants, and of these 20 had received all outreaches, with the remaining 62 participants being at differing stages of the intervention timeline. All participants had received at least 2 of 7 outreaches via the automated calls before premature study completion. We therefore modified the intervention, and the call content for the remaining outreaches was delivered only via survey format. This survey (delivered via REDCap) contained replicated content and questions as asked in the phone calls but required participants to click their responses. The hope had been to find another technology partner that could deliver the intervention according to our specifications, but we were unable to do this and limited by the remaining budget, we stopped the study to report findings. These changes were planned, reviewed, and approved by the trial steering committee. This trial is reported according to the CONSERVE (CONSORT and SPIRIT Extension for RCTs Revised in Extenuating Circumstances) statement[18]."

8a) Method used to generate the random allocation sequence
NPT: When applicable, how care providers were allocated to each trial group

Does your paper address CONSORT subitem 8a? *

Copy and paste relevant sections from the manuscript (include quotes in quotation marks "like this" to indicate direct quotes from your manuscript), or elaborate on this item by providing additional information not in the ms, or briefly explain why the item is not applicable/relevant for your study

Details regarding randomisation are outlined in the methods - "Randomisation was 4:1 (intervention: control), stratified by sex, in blocks of 5. The allocation sequence was incorporated into Research Electronic Data Capture (REDCap)[16] with access groups enabled, where data analysts were blinded. Participants, care providers, and research assistants were not blinded. Participants were randomised to receive usual care or the conversational AI intervention."

8b) Type of randomisation; details of any restriction (such as blocking and block size)

Does your paper address CONSORT subitem 8b? *

Copy and paste relevant sections from the manuscript (include quotes in quotation marks "like this" to indicate direct quotes from your manuscript), or elaborate on this item by providing additional information not in the ms, or briefly explain why the item is not applicable/relevant for your study

Details regarding randomisation are outlined in the methods - "Randomisation was 4:1 (intervention: control), stratified by sex, in blocks of 5. The allocation sequence was incorporated into Research Electronic Data Capture (REDCap)[16] with access groups enabled, where data analysts were blinded. Participants, care providers, and research assistants were not blinded. Participants were randomised to receive usual care or the conversational AI intervention."

9) Mechanism used to implement the random allocation sequence (such as sequentially numbered containers), describing any steps taken to conceal the sequence until interventions were assigned

Does your paper address CONSORT subitem 9? *

Copy and paste relevant sections from the manuscript (include quotes in quotation marks "like this" to indicate direct quotes from your manuscript), or elaborate on this item by providing additional information not in the ms, or briefly explain why the item is not applicable/relevant for your study

Details regarding randomisation are outlined in the methods - "Randomisation was 4:1 (intervention: control), stratified by sex, in blocks of 5. The allocation sequence was incorporated into Research Electronic Data Capture (REDCap)[16] with access groups enabled, where data analysts were blinded. Participants, care providers, and research assistants were not blinded. Participants were randomised to receive usual care or the conversational AI intervention."

10) Who generated the random allocation sequence, who enrolled participants, and who assigned participants to interventions

Does your paper address CONSORT subitem 10? *

Copy and paste relevant sections from the manuscript (include quotes in quotation marks "like this" to indicate direct quotes from your manuscript), or elaborate on this item by providing additional information not in the ms, or briefly explain why the item is not applicable/relevant for your study

A blinded statistician generated the randomisation allocation sequence and uploaded it onto REDCap. This allocation sequence was not accessible to those who enrolled study participants. Details regarding randomisation are outlined in the methods - "Randomisation was 4:1 (intervention: control), stratified by sex, in blocks of 5. The allocation sequence was incorporated into Research Electronic Data Capture (REDCap)[16] with access groups enabled, where data analysts were blinded. Participants, care providers, and research assistants were not blinded. Participants were randomised to receive usual care or the conversational AI intervention."

11a) If done, who was blinded after assignment to interventions (for example, participants, care providers, those assessing outcomes) and how

NPT: Whether or not administering co-interventions were blinded to group assignment

11a-i) Specify who was blinded, and who wasn't

Specify who was blinded, and who wasn't. Usually, in web-based trials it is not possible to blind the participants [1, 3] (this should be clearly acknowledged), but it may be possible to blind outcome assessors, those doing data analysis or those administering co-interventions (if any).

	1	2	3	4	5	
subitem not at all important	○	○	○	○	○	essential

Does your paper address subitem 11a-i? *

Copy and paste relevant sections from the manuscript (include quotes in quotation marks "like this" to indicate direct quotes from your manuscript), or elaborate on this item by providing additional information not in the ms, or briefly explain why the item is not applicable/relevant for your study

Details regarding randomisaton and blinding are outlined in the methods - "Randomisation was 4:1 (intervention: control), stratified by sex, in blocks of 5. The allocation sequence was incorporated into Research Electronic Data Capture (REDCap)[16] with access groups enabled, where data analysts were blinded. Participants, care providers, and research assistants were not blinded. Participants were randomised to receive usual care or the conversational AI intervention."

11a-ii) Discuss e.g., whether participants knew which intervention was the “intervention of interest” and which one was the “comparator”

Informed consent procedures (4a-ii) can create biases and certain expectations - discuss e.g., whether participants knew which intervention was the “intervention of interest” and which one was the “comparator”.

	1	2	3	4	5	
subitem not at all important	○	○	○	○	○	essential

Does your paper address subitem 11a-ii?

Copy and paste relevant sections from the manuscript (include quotes in quotation marks "like this" to indicate direct quotes from your manuscript), or elaborate on this item by providing additional information not in the ms, or briefly explain why the item is not applicable/relevant for your study

Your answer

11b) If relevant, description of the similarity of interventions

(this item is usually not relevant for ehealth trials as it refers to similarity of a placebo or sham intervention to a active medication/intervention)

Does your paper address CONSORT subitem 11b? *

Copy and paste relevant sections from the manuscript (include quotes in quotation marks "like this" to indicate direct quotes from your manuscript), or elaborate on this item by providing additional information not in the ms, or briefly explain why the item is not applicable/relevant for your study

Not relevant to the current study

12a) Statistical methods used to compare groups for primary and secondary outcomes

NPT: When applicable, details of whether and how the clustering by care providers or centers was addressed

Does your paper address CONSORT subitem 12a? *

Copy and paste relevant sections from the manuscript (include quotes in quotation marks "like this" to indicate direct quotes from your manuscript), or elaborate on this item by providing additional information not in the ms, or briefly explain why the item is not applicable/relevant for your study

Statistical methods are described in the methods section - "Analyses were pre-specified in a statistical analysis plan and were conducted according to intention-to-treat principles. Analyses were performed using R statistical software version 4.1.2 (R Project for Statistical Computing). Outcomes were analysed using either ANCOVA or logistic regression, adjusting for baseline variables. All tests were two-tailed, a p-value of <0.05 was considered significant and odds ratios (OR) were reported with 95% confidence intervals (CI). Normally distributed continuous variables were expressed as mean and standard deviation (SD). Non-normally distributed variables were expressed as the median and interquartile range (IQR)."

12a-i) Imputation techniques to deal with attrition / missing values

Imputation techniques to deal with attrition / missing values: Not all participants will use the intervention/comparator as intended and attrition is typically high in ehealth trials. Specify how participants who did not use the application or dropped out from the trial were treated in the statistical analysis (a complete case analysis is strongly discouraged, and simple imputation techniques such as LOCF may also be problematic [4]).

	1	2	3	4	5	
subitem not at all important	○	○	○	○	○	essential

Does your paper address subitem 12a-i? *

Copy and paste relevant sections from the manuscript (include quotes in quotation marks "like this" to indicate direct quotes from your manuscript), or elaborate on this item by providing additional information not in the ms, or briefly explain why the item is not applicable/relevant for your study

Information on dealing with missing values is provided in the manuscript - "There were 18.4% missing primary outcome data at 6 months, however, we found no evidence for differences in missingness based on age (grouped by 65 years or older), gender, or ethnicity (grouped by Caucasian or non-Caucasian). Pre-specified sensitivity analyses were conducted, both imputation of 3-month AFEQT total score carried forward and baseline imputation revealed similar results to the primary analysis."

12b) Methods for additional analyses, such as subgroup analyses and adjusted analyses

Does your paper address CONSORT subitem 12b? *

Copy and paste relevant sections from the manuscript (include quotes in quotation marks "like this" to indicate direct quotes from your manuscript), or elaborate on this item by providing additional information not in the ms, or briefly explain why the item is not applicable/relevant for your study

Statistical methods are described in the methods section - "Analyses were pre-specified in a statistical analysis plan and were conducted according to intention-to-treat principles. Analyses were performed using R statistical software version 4.1.2 (R Project for Statistical Computing). Outcomes were analysed using either ANCOVA or logistic regression, adjusting for baseline variables. All tests were two-tailed, a p-value of <0.05 was considered significant and odds ratios (OR) were reported with 95% confidence intervals (CI). Normally distributed continuous variables were expressed as mean and standard deviation (SD). Non-normally distributed variables were expressed as the median and interquartile range (IQR). A univariate logistic regression analysis to predict high engagement was conducted with covariates of age, gender, ethnicity, education, type of AF, time since AF diagnosis, and CHAD2S2-VASc score (Congestive heart failure, hypertension, age 75 years and above (2 points), diabetes, stroke (2 points), vascular disease, age 65 to 74 and sex category (female); calculated as a sum, where 1-point or 2-points (where indicated) is given when aforementioned characteristics are present)[24]"

X26) REB/IRB Approval and Ethical Considerations [recommended as subheading under "Methods"] (not a CONSORT item)

X26-i) Comment on ethics committee approval

	1	2	3	4	5	
subitem not at all important	○	○	○	○	○	essential

Does your paper address subitem X26-i?

Copy and paste relevant sections from the manuscript (include quotes in quotation marks "like this" to indicate direct quotes from your manuscript), or elaborate on this item by providing additional information not in the ms, or briefly explain why the item is not applicable/relevant for your study

Ethics approval was obtained - "The ethics committee at Western Sydney Local Health District (2020/ETH02546) granted approval."

x26-ii) Outline informed consent procedures

Outline informed consent procedures e.g., if consent was obtained offline or online (how? Checkbox, etc.?), and what information was provided (see 4a-ii). See [6] for some items to be included in informed consent documents.

	1	2	3	4	5	
subitem not at all important	○	○	○	○	○	essential

Does your paper address subitem X26-ii?

Copy and paste relevant sections from the manuscript (include quotes in quotation marks "like this" to indicate direct quotes from your manuscript), or elaborate on this item by providing additional information not in the ms, or briefly explain why the item is not applicable/relevant for your study

Information regarding consent is provided in the manuscript - "All participants provided informed consent (written or phone consent) and completed study assessments via electronic surveys at baseline (in hospital or sent link via text messages/email), 3- and 6-months (via link sent by text message or email)."

X26-iii) Safety and security procedures

Safety and security procedures, incl. privacy considerations, and any steps taken to reduce the likelihood or detection of harm (e.g., education and training, availability of a hotline)

	1	2	3	4	5	
subitem not at all important	○	○	○	○	○	essential

Does your paper address subitem X26-iii?

Copy and paste relevant sections from the manuscript (include quotes in quotation marks "like this" to indicate direct quotes from your manuscript), or elaborate on this item by providing additional information not in the ms, or briefly explain why the item is not applicable/relevant for your study

A study email and phone number were available for participants to contact the research team.

RESULTS

13a) For each group, the numbers of participants who were randomly assigned, received intended treatment, and were analysed for the primary outcome
NPT: The number of care providers or centers performing the intervention in each group and the number of patients treated by each care provider in each center

Does your paper address CONSORT subitem 13a? *

Copy and paste relevant sections from the manuscript (include quotes in quotation marks "like this" to indicate direct quotes from your manuscript), or elaborate on this item by providing additional information not in the ms, or briefly explain why the item is not applicable/relevant for your study

This is provided in Figure 1.

13b) For each group, losses and exclusions after randomisation, together with reasons

Does your paper address CONSORT subitem 13b? (NOTE: Preferably, this is shown in a CONSORT flow diagram) *

Copy and paste relevant sections from the manuscript (include quotes in quotation marks "like this" to indicate direct quotes from your manuscript), or elaborate on this item by providing additional information not in the ms, or briefly explain why the item is not applicable/relevant for your study

This is provided in Figure 1.

13b-i) Attrition diagram

Strongly recommended: An attrition diagram (e.g., proportion of participants still logging in or using the intervention/comparator in each group plotted over time, similar to a survival curve) or other figures or tables demonstrating usage/dose/engagement.

	1	2	3	4	5	
subitem not at all important	○	○	○	○	○	essential

Does your paper address subitem 13b-i?

Copy and paste relevant sections from the manuscript or cite the figure number if applicable (include quotes in quotation marks "like this" to indicate direct quotes from your manuscript), or elaborate on this item by providing additional information not in the ms, or briefly explain why the item is not applicable/relevant for your study

This is provided in Figure 3a and in Multimedia Appendix 1.

14a) Dates defining the periods of recruitment and follow-up

Does your paper address CONSORT subitem 14a? *

Copy and paste relevant sections from the manuscript (include quotes in quotation marks "like this" to indicate direct quotes from your manuscript), or elaborate on this item by providing additional information not in the ms, or briefly explain why the item is not applicable/relevant for your study

This information is provided in the results - "Between January to July 2021, we enrolled 103 participants (82 intervention, 21 control) (Figure 1). We lost 16 intervention participants and 3 controls to follow-up (19.4%), and the primary outcome analysis included 66 intervention participants and 19 controls. Follow-up period was from July 2021 to April 2022."

14a-i) Indicate if critical “secular events” fell into the study period

Indicate if critical “secular events” fell into the study period, e.g., significant changes in Internet resources available or “changes in computer hardware or Internet delivery resources”

	1	2	3	4	5	
subitem not at all important	○	○	○	○	○	essential

Does your paper address subitem 14a-i?

Copy and paste relevant sections from the manuscript (include quotes in quotation marks "like this" to indicate direct quotes from your manuscript), or elaborate on this item by providing additional information not in the ms, or briefly explain why the item is not applicable/relevant for your study

Changes to intervention delivery occurred during the study period and this has been explained in detail - "At the end of July 2021, we stopped recruitment as the delivery of the intervention was interrupted due to unforeseeable circumstances involving acquisition of the technology partner by another organisation. We had already recruited 82 intervention participants, and of these 20 had received all outreaches, with the remaining 62 participants being at differing stages of the intervention timeline. All participants had received at least 2 of 7 outreaches via the automated calls before premature study completion. We therefore modified the intervention, and the call content for the remaining outreaches was delivered only via survey format. This survey (delivered via REDCap) contained replicated content and questions as asked in the phone calls but required participants to click their responses. The hope had been to find another technology partner that could deliver the intervention according to our specifications, but we were unable to do this and limited by the remaining budget, we stopped the study to report findings. These changes were planned, reviewed, and approved by the trial steering committee. This trial is reported according to the CONSERVE (CONSORT and SPIRIT Extension for RCTs Revised in Extenuating Circumstances) statement[18]."

14b) Why the trial ended or was stopped (early)

Does your paper address CONSORT subitem 14b? *

Copy and paste relevant sections from the manuscript (include quotes in quotation marks "like this" to indicate direct quotes from your manuscript), or elaborate on this item by providing additional information not in the ms, or briefly explain why the item is not applicable/relevant for your study

The trial was stopped early and this has been explained in detail in the manuscript - "At the end of July 2021, we stopped recruitment as the delivery of the intervention was interrupted due to unforeseeable circumstances involving acquisition of the technology partner by another organisation. We had already recruited 82 intervention participants, and of these 20 had received all outreaches, with the remaining 62 participants being at differing stages of the intervention timeline. All participants had received at least 2 of 7 outreaches via the automated calls before premature study completion. We therefore modified the intervention, and the call content for the remaining outreaches was delivered only via survey format. This survey (delivered via REDCap) contained replicated content and questions as asked in the phone calls but required participants to click their responses. The hope had been to find another technology partner that could deliver the intervention according to our specifications, but we were unable to do this and limited by the remaining budget, we stopped the study to report findings. These changes were planned, reviewed, and approved by the trial steering committee. This trial is reported according to the CONSERVE (CONSORT and SPIRIT Extension for RCTs Revised in Extenuating Circumstances) statement[18]."

15) A table showing baseline demographic and clinical characteristics for each group

NPT: When applicable, a description of care providers (case volume, qualification, expertise, etc.) and centers (volume) in each group

Does your paper address CONSORT subitem 15? *

Copy and paste relevant sections from the manuscript (include quotes in quotation marks "like this" to indicate direct quotes from your manuscript), or elaborate on this item by providing additional information not in the ms, or briefly explain why the item is not applicable/relevant for your study

This is provided in Table 1 and in the Multimedia Appendix 1.

15-i) Report demographics associated with digital divide issues

In ehealth trials it is particularly important to report demographics associated with digital divide issues, such as age, education, gender, social-economic status, computer/Internet/ehealth literacy of the participants, if known.

	1	2	3	4	5	
subitem not at all important	○	○	○	○	○	essential

Does your paper address subitem 15-i? *

Copy and paste relevant sections from the manuscript (include quotes in quotation marks "like this" to indicate direct quotes from your manuscript), or elaborate on this item by providing additional information not in the ms, or briefly explain why the item is not applicable/relevant for your study

This is provided in Table 1 and in the Multimedia Appendix 1.

16) For each group, number of participants (denominator) included in each analysis and whether the analysis was by original assigned groups

16-i) Report multiple “denominators” and provide definitions

Report multiple “denominators” and provide definitions: Report N’s (and effect sizes) “across a range of study participation [and use] thresholds” [1], e.g., N exposed, N consented, N used more than x times, N used more than y weeks, N participants “used” the intervention/comparator at specific pre-defined time points of interest (in absolute and relative numbers per group). Always clearly define “use” of the intervention.

	1	2	3	4	5	
subitem not at all important	○	○	○	○	○	essential

Does your paper address subitem 16-i? *

Copy and paste relevant sections from the manuscript (include quotes in quotation marks "like this" to indicate direct quotes from your manuscript), or elaborate on this item by providing additional information not in the ms, or briefly explain why the item is not applicable/relevant for your study

All tables include a n= value (denominator).

16-ii) Primary analysis should be intent-to-treat

Primary analysis should be intent-to-treat, secondary analyses could include comparing only "users", with the appropriate caveats that this is no longer a randomized sample (see 18-i).

	1	2	3	4	5	
subitem not at all important	○	○	○	○	○	essential

Does your paper address subitem 16-ii?

Copy and paste relevant sections from the manuscript (include quotes in quotation marks "like this" to indicate direct quotes from your manuscript), or elaborate on this item by providing additional information not in the ms, or briefly explain why the item is not applicable/relevant for your study

Primary analysis was intention-to-treat.

17a) For each primary and secondary outcome, results for each group, and the estimated effect size and its precision (such as 95% confidence interval)

Does your paper address CONSORT subitem 17a? *

Copy and paste relevant sections from the manuscript (include quotes in quotation marks "like this" to indicate direct quotes from your manuscript), or elaborate on this item by providing additional information not in the ms, or briefly explain why the item is not applicable/relevant for your study

Results from primary and secondary outcome analysis have been displaying in Table 2, Figure 2 and Figure 3, and additional data is available in the online Multimedia Appendix 1.

17a-i) Presentation of process outcomes such as metrics of use and intensity of use

In addition to primary/secondary (clinical) outcomes, the presentation of process outcomes such as metrics of use and intensity of use (dose, exposure) and their operational definitions is critical. This does not only refer to metrics of attrition (13-b) (often a binary variable), but also to more continuous exposure metrics such as "average session length". These must be accompanied by a technical description how a metric like a "session" is defined (e.g., timeout after idle time) [1] (report under item 6a).

	1	2	3	4	5	
subitem not at all important	○	○	○	○	○	essential

Does your paper address subitem 17a-i?

Copy and paste relevant sections from the manuscript (include quotes in quotation marks "like this" to indicate direct quotes from your manuscript), or elaborate on this item by providing additional information not in the ms, or briefly explain why the item is not applicable/relevant for your study

Process evaluation outcomes are presented in Figure 3 and additional data is available in the online Multimedia Appendix 1.

17b) For binary outcomes, presentation of both absolute and relative effect sizes is recommended

Does your paper address CONSORT subitem 17b? *

Copy and paste relevant sections from the manuscript (include quotes in quotation marks "like this" to indicate direct quotes from your manuscript), or elaborate on this item by providing additional information not in the ms, or briefly explain why the item is not applicable/relevant for your study

Results from primary and secondary outcome analysis have been displaying in Table 2, Figure 2 and Figure 3, and additional data is available in the online Multimedia Appendix 1.

18) Results of any other analyses performed, including subgroup analyses and adjusted analyses, distinguishing pre-specified from exploratory

Does your paper address CONSORT subitem 18? *

Copy and paste relevant sections from the manuscript (include quotes in quotation marks "like this" to indicate direct quotes from your manuscript), or elaborate on this item by providing additional information not in the ms, or briefly explain why the item is not applicable/relevant for your study

All analysis were pre-specified in a statistical analysis plan, however, exploratory primary outcome analysis was undertaken, and this has been outlined in the results section - "Additional exploratory analyses revealed a significant improvement in AFEQT total score post-intervention in the intervention group (baseline = 69.9, 95% CI: 64.4–75.5; 6-months = 79.9, 95% CI: 74.9–84.8; p=0.01), with no improvements in the control group (Figure 2). Within-group differences revealed the intervention group had a significant improvement in most AFEQT subscales post-intervention (symptom, daily activity, treatment), with no improvements seen in the control group (Figure 2) (Table 2)."

18-i) Subgroup analysis of comparing only users

A subgroup analysis of comparing only users is not uncommon in ehealth trials, but if done, it must be stressed that this is a self-selected sample and no longer an unbiased sample from a randomized trial (see 16-iii).

	1	2	3	4	5	
subitem not at all important	○	○	○	○	○	essential

Does your paper address subitem 18-i?

Copy and paste relevant sections from the manuscript (include quotes in quotation marks "like this" to indicate direct quotes from your manuscript), or elaborate on this item by providing additional information not in the ms, or briefly explain why the item is not applicable/relevant for your study

Your answer

19) All important harms or unintended effects in each group

(for specific guidance see CONSORT for harms)

Does your paper address CONSORT subitem 19? *

Copy and paste relevant sections from the manuscript (include quotes in quotation marks "like this" to indicate direct quotes from your manuscript), or elaborate on this item by providing additional information not in the ms, or briefly explain why the item is not applicable/relevant for your study

There were no harms or unintended effects.

19-i) Include privacy breaches, technical problems

Include privacy breaches, technical problems. This does not only include physical “harm” to participants, but also incidents such as perceived or real privacy breaches [1], technical problems, and other unexpected/unintended incidents. “Unintended effects” also includes unintended positive effects [2].

	1	2	3	4	5	
subitem not at all important	○	○	○	○	○	essential

Does your paper address subitem 19-i?

Copy and paste relevant sections from the manuscript (include quotes in quotation marks "like this" to indicate direct quotes from your manuscript), or elaborate on this item by providing additional information not in the ms, or briefly explain why the item is not applicable/relevant for your study

Your answer

19-ii) Include qualitative feedback from participants or observations from staff/researchers

Include qualitative feedback from participants or observations from staff/researchers, if available, on strengths and shortcomings of the application, especially if they point to unintended/unexpected effects or uses. This includes (if available) reasons for why people did or did not use the application as intended by the developers.

	1	2	3	4	5	
subitem not at all important	○	○	○	○	○	essential

Does your paper address subitem 19-ii?

Copy and paste relevant sections from the manuscript (include quotes in quotation marks "like this" to indicate direct quotes from your manuscript), or elaborate on this item by providing additional information not in the ms, or briefly explain why the item is not applicable/relevant for your study

Your answer

DISCUSSION

22) Interpretation consistent with results, balancing benefits and harms, and considering other relevant evidence

NPT: In addition, take into account the choice of the comparator, lack of or partial blinding, and unequal expertise of care providers or centers in each group

22-i) Restate study questions and summarize the answers suggested by the data, starting with primary outcomes and process outcomes (use)

Restate study questions and summarize the answers suggested by the data, starting with primary outcomes and process outcomes (use).

	1	2	3	4	5	
subitem not at all important	○	○	○	○	○	essential

Does your paper address subitem 22-i? *

Copy and paste relevant sections from the manuscript (include quotes in quotation marks "like this" to indicate direct quotes from your manuscript), or elaborate on this item by providing additional information not in the ms, or briefly explain why the item is not applicable/relevant for your study

The principal results have been summarised in the discussion - "The CHAT-AF study provides a proof-of-concept with initial data on the efficacy of a novel digital health follow-up strategy for AF patients that leverages conversational AI. Its demonstration of high levels of engagement and satisfaction with the digital outreaches suggests this approach to delivering care is feasible and could enable chronic disease services to follow up with patients at scale with fewer in-person and staffed visits. Due to challenges with the technology partner acquisition, the study had only recruited 27% of its intended sample size and was subsequently underpowered for its primary outcome. The trial did not demonstrate a significant difference in the primary outcome between intervention and control, however, there was a benefit suggested by an exploratory pre-post comparison that showed overall AF-QoL improved at 6 months in the intervention group from baseline, with no similar improvement observed in the control group."

22-ii) Highlight unanswered new questions, suggest future research

Highlight unanswered new questions, suggest future research.

	1	2	3	4	5	
subitem not at all important	○	○	○	○	○	essential

Does your paper address subitem 22-ii?

Copy and paste relevant sections from the manuscript (include quotes in quotation marks "like this" to indicate direct quotes from your manuscript), or elaborate on this item by providing additional information not in the ms, or briefly explain why the item is not applicable/relevant for your study

Your answer

20) Trial limitations, addressing sources of potential bias, imprecision, and, if relevant, multiplicity of analyses

20-i) Typical limitations in ehealth trials

Typical limitations in ehealth trials: Participants in ehealth trials are rarely blinded. Ehealth trials often look at a multiplicity of outcomes, increasing risk for a Type I error. Discuss biases due to non-use of the intervention/usability issues, biases through informed consent procedures, unexpected events.

	1	2	3	4	5	
subitem not at all important	○	○	○	○	○	essential

Does your paper address subitem 20-i? *

Copy and paste relevant sections from the manuscript (include quotes in quotation marks "like this" to indicate direct quotes from your manuscript), or elaborate on this item by providing additional information not in the ms, or briefly explain why the item is not applicable/relevant for your study

Trial limitations are mentioned in the discussion - "This study has several limitations which need to be considered. The current study was limited in not achieving its target sample size and multi-centre reach due to premature study completion and therefore careful consideration needs to be made when interpreting the results as the study was underpowered. This also meant some participants on the intervention arm were unable to complete their conversational AI 'visits' later into the planned follow-up period which is likely to have contributed to the ability to evaluate the effectiveness of the intervention on outcomes. Moreover, except for baseline characteristics (medical history), all outcome data was self-reported. There was a difference in attrition between the intervention (16/82) and control (3/21) groups which may have impacted the primary analysis, however, missingness was checked for and we found no differences in terms of age, gender, or ethnicity. The study population was younger (63.7±11.2 years) and had a lower proportion of women (30.1%) than previously reported literature of the AF patient population in Australia (mean age of 75 years and approximately 45% female)[32]. The study was delivered in English and included predominately Caucasian participants, highlighting the need for further work to validate this technology in larger trials with more diverse and representative patient populations."

21) Generalisability (external validity, applicability) of the trial findings

NPT: External validity of the trial findings according to the intervention, comparators, patients, and care providers or centers involved in the trial

21-i) Generalizability to other populations

Generalizability to other populations: In particular, discuss generalizability to a general Internet population, outside of a RCT setting, and general patient population, including applicability of the study results for other organizations

	1	2	3	4	5	
subitem not at all important	○	○	○	○	○	essential

Does your paper address subitem 21-i?

Copy and paste relevant sections from the manuscript (include quotes in quotation marks "like this" to indicate direct quotes from your manuscript), or elaborate on this item by providing additional information not in the ms, or briefly explain why the item is not applicable/relevant for your study

Your answer

21-ii) Discuss if there were elements in the RCT that would be different in a routine application setting

Discuss if there were elements in the RCT that would be different in a routine application setting (e.g., prompts/reminders, more human involvement, training sessions or other co-interventions) and what impact the omission of these elements could have on use, adoption, or outcomes if the intervention is applied outside of a RCT setting.

	1	2	3	4	5	
subitem not at all important	○	○	○	○	○	essential

Does your paper address subitem 21-ii?

Copy and paste relevant sections from the manuscript (include quotes in quotation marks "like this" to indicate direct quotes from your manuscript), or elaborate on this item by providing additional information not in the ms, or briefly explain why the item is not applicable/relevant for your study

Your answer

OTHER INFORMATION

23) Registration number and name of trial registry

Does your paper address CONSORT subitem 23? *

Copy and paste relevant sections from the manuscript (include quotes in quotation marks "like this" to indicate direct quotes from your manuscript), or elaborate on this item by providing additional information not in the ms, or briefly explain why the item is not applicable/relevant for your study

Australian New Zealand Clinical Trials Registry (ACTRN12621000174886)

24) Where the full trial protocol can be accessed, if available

Does your paper address CONSORT subitem 24? *

Cite a Multimedia Appendix, other reference, or copy and paste relevant sections from the manuscript (include quotes in quotation marks "like this" to indicate direct quotes from your manuscript), or elaborate on this item by providing additional information not in the ms, or briefly explain why the item is not applicable/relevant for your study

Protocol published in JMIR Research Protocols -
https://researchprotocols.org/2022/4/e34470/

25) Sources of funding and other support (such as supply of drugs), role of funders

Does your paper address CONSORT subitem 25? *

Copy and paste relevant sections from the manuscript (include quotes in quotation marks "like this" to indicate direct quotes from your manuscript), or elaborate on this item by providing additional information not in the ms, or briefly explain why the item is not applicable/relevant for your study

This work is supported by Digital Health CRC Limited (DHCRC). DHCRC is funded under the Commonwealth's Cooperative Research Centres (CRC) Program.

X27) Conflicts of Interest (not a CONSORT item)

X27-i) State the relation of the study team towards the system being evaluated

In addition to the usual declaration of interests (financial or otherwise), also state the relation of the study team towards the system being evaluated, i.e., state if the authors/evaluators are distinct from or identical with the developers/sponsors of the intervention.

	1	2	3	4	5	
subitem not at all important	○	○	○	○	○	essential

Does your paper address subitem X27-i?

Copy and paste relevant sections from the manuscript (include quotes in quotation marks "like this" to indicate direct quotes from your manuscript), or elaborate on this item by providing additional information not in the ms, or briefly explain why the item is not applicable/relevant for your study

No conflicts of interest to declare.

About the CONSORT EHEALTH checklist

As a result of using this checklist, did you make changes in your manuscript? *

- ( ) yes, major changes
- (x) yes, minor changes
- ( ) no

What were the most important changes you made as a result of using this checklist?

Your answer

How much time did you spend on going through the checklist INCLUDING making changes in your manuscript *

A total of 2.5 hours.

Your answer must have a minimum of 25 characters.

As a result of using this checklist, do you think your manuscript has improved? *

- (x) yes
- ( ) no
- ( ) Other:

Would you like to become involved in the CONSORT EHEALTH group?

This would involve for example becoming involved in participating in a workshop and writing an "Explanation and Elaboration" document

- yes
- no
- Other:

Any other comments or questions on CONSORT EHEALTH

Your answer

STOP - Save this form as PDF before you click submit

To generate a record that you filled in this form, we recommend to generate a PDF of this page (on a Mac, simply select "print" and then select "print as PDF") before you submit it.

When you submit your (revised) paper to JMIR, please upload the PDF as supplementary file.

Don't worry if some text in the textboxes is cut off, as we still have the complete information in our database. Thank you!

Final step: Click submit !

Click submit so we have your answers in our database!

Submit Clear form

Never submit passwords through Google Forms.

This content is neither created nor endorsed by Google. Report Abuse - Terms of Service - Privacy Policy

Google Forms
